# Density Functionals of Chemical Bonding

**DOI:** 10.3390/ijms9061050

**Published:** 2008-06-26

**Authors:** Mihai V. Putz

**Affiliations:** Laboratory of Computational and Structural Physical Chemistry, Chemistry Department, West University of Timişoara, Pestalozzi Street No.16, Timişoara, RO-300115, Romania E-mails: mvputz@cbg.uvt.ro or mv_putz@yahoo.com; Web: www.cbg.uvt.ro/mvputz

**Keywords:** density functional theory, electronic localization function, kinetic energy, exchange energy, correlation energy, exchange-correlation energy, electronegativity, chemical hardness, QSPR

## Abstract

The behavior of electrons in general many-electronic systems throughout the density functionals of energy is reviewed. The basic physico-chemical concepts of density functional theory are employed to highlight the energy role in chemical structure while its extended influence in electronic localization function helps in chemical bonding understanding. In this context the energy functionals accompanied by electronic localization functions may provide a comprehensive description of the global-local levels electronic structures in general and of chemical bonds in special. Becke-Edgecombe and author’s Markovian electronic localization functions are discussed at atomic, molecular and solid state levels. Then, the analytical survey of the main workable kinetic, exchange, and correlation density functionals within local and gradient density approximations is undertaken. The hierarchy of various energy functionals is formulated by employing both the parabolic and statistical correlation degree of them with the electronegativity and chemical hardness indices by means of quantitative structure-property relationship (QSPR) analysis for basic atomic and molecular systems.

## 1. Introduction

In Walter Kohn’s lecture, with the occasion of receiving his Nobel Prize in Chemistry [1], back in 1998, for density functional theory (DFT) theory [2–4], there was formulated a quite provoking assertion affirming that, *heuristically, the general eigen-wave-function* Ψ (**r**_1_,...,**r***_N_* ) *associated to a system of N electrons fails to be a legitimate scientific concept when N* ≥ *N*_0_ ≈ 10^3^.

Nevertheless, this affirmation may be at any time turned in a theorem, eventually as *Kohn’s zero DFT theorem*, with a proof following the van Vleck prescription of the so called „exponential wall”, leaving with the applicability limits of the conceptually eigen-wave function of multi-electronics systems. However, before proceeding to demonstration, there must be noted that such reality limitation characterized by eigen-wave-function of the multi electronic-systems is not transferable at the quantum mechanics postulates, but providing an alternative quantum scheme, thus paralleling Schrödinger equation, however in a more generally integrated level.

The demonstration of the non-representability of the eigen-function for systems containing more than *N*_0_ ≈ 10^3^ electrons involves two aspects: *the accuracy of representation* by using the eigen-wave function and the *possibility of measuring it*.

Regarding the accuracy of the Ψ(**r**_1_,...,**r***_N_*) representation there is widely known that it associates with the density of probability smoothly approaching unity, written in the “liberal” form (according to Kohn):

(1) $$|{\text{Ψ}}^{*}({\text{r}}_{1},...{\text{r}}_{N})\text{Ψ}({\text{r}}_{1},...,{\text{r}}_{N})|=1-ɛ\;,\;ɛ=10^{-2}$$

Now, considering a collection of *N’* molecules the total density of probability of this multi-molecular system (and implicit a multi-electronic one) will consequently be:

(2) $$\left|{\left({\text{Ψ}}^{*}\text{Ψ}\right)}_{1}...{\left({\text{Ψ}}^{*}\text{Ψ}\right)}_{N'}\right|={\left(1-ɛ\right)}^{N'}\;\underset{=}{∼}\;\exp(-ɛN')=\exp[-10^{-2}N'].$$

For *N’*=10^3^ molecules in whatever aggregates, e.g. solids, clusters, super-molecules, or biological macro-molecules, the total density of probability will result from (2) as exp[−10]≈5×10^−5^, meaning that it is determined with much less accuracy compared with the degree of individual eigen-function localization precision in (1). Since each molecular system has at least one electron there follows the threshold limit of *N_0_* ≈ *10^3^* electrons from where the lost in associated wavefunction nature is recorded.

This result, relaying on the exponential form (2), justifies the title of „exponential wall” for the wavefunction limitation.

Then, going to the measurable issue of such eigenfunctions, let’s ask how many bits are necessary for recording its quantum dimension? Assume, again, the working wavefunction Ψ(**r**_1_,...,**r***_N_*) for all the *N* electrons in a concerned system. The *N* electrons in system have a total of 3*N* space variables (in the configuration space); let’s now assume an average of *q* bits necessary in measuring a single variable from the total of 3*N*; there results a total information of

(3) $$B=q^{3N}$$

bits for recording (storing) the total eigen-wave-function of the system. However, a simple evaluation of the dimension (3) shows that for a minimum of *q*=3 bits/variable and for the above consecrated minimum limit of *N=N*_0_≈10^3^ electrons in the system the total yield of necessary bits for recording is about of 10^1000^ order – a truly non-realistic dimension. This can be immediately visualized if one recalls that the *total number of baryons* (i.e. all fermions and elementary particles of protonic and neutronic type, but not limited only to these) *estimated in entirely Universe* (summing up all existing atoms and free nuclei in the plasma state, but not only limited to these) *gives a result of about* 10^80^ order.

Definitely, the concept of eigen-wave function must be enlarged or modified in such a manner that the quantum description does not be blocked by the exponential wall: from where we can start? Firstly, as was exposed, the eigen-wave function in the configuration space multiplies in an exponential manner the variables accounting for the number and the position of the electrons; thus, the configuration space must be avoided. Then, the density of probability must be reformulated as such the exponential wall for a poly-electronic system be avoided while preserving the dependency of the total number of electrons *N*.

Fortunately, the above described conceptual project was unfolded in 1963 when Walter Kohn met in Paris (at École Normale Supérieure), during his sabbatical semester, the mate Pierre Hohenberg who was working at the description of the metallic alloys (specially the Cu_x_Zn_1-x_ systems) by using quantum traditionally methods of averaging crystalline periodic field. Studies of this type of problems often start from the level of the uniform electronic density referential upon which specific perturbation treatments are applied. From this point Kohn and Hohenberg made two crucial further steps in reformulation of the quantum picture of the matter structure: one referred at the electronic density, and another at the relation between electronic density with the externally applied potential on the electronic system; they were consecrated in the so called Hohenberg-Kohn (KH) theorems [2, 5–7]. They were the fundaments of new emerging quantum density functional theory that mostly impacted the reformation of the quantum chemistry itself and its foreground principles of structure and transformation.

The present work likes to review some fundamental aspects of density functional theory highlighting on the primer conceptual and computational consequences in electronic localization and chemical reactivity.

## 2. Primary Density Functional Theory Concepts

### 2.1. Hohenberg-Kohn theorems

The first Hohenberg-Kohn (HK1) theorem gives space to the concept of *electronic density of the system* ρ(**r**) in terms of the extensive relation with the *N* electrons from the system that it characterizes [8]:

(4) ∫ρ(r)dr=N  .

The relation (4) as much simple it could appears stands as the decisive passage from the eigen-wave function level to the level of total electronic density [9–11]:

(5) $$\rho (\text{r})=N\int {\text{Ψ}}^{*}(\text{r},{\text{r}}_{2},...,{\text{r}}_{N})\text{Ψ}(\text{r},{\text{r}}_{2},...,{\text{r}}_{N})d{\text{r}}_{2}...d{\text{r}}_{N}.$$

Firstly, Eq. (5) satisfies Eq. (4); this can be used also as simple immediate proof of the relation (4) itself. Then, the dependency from the 3*N*-dimensions of configuration space was reduced at 3 coordinates in the real space, physically measurable.

However, still remains the question: what represents the electronic density of Eq. (5)? Definitely, it neither represents the electronic density in the configuration space nor the density of a single electron, since the *N*-electronic dependency as multiplication factor of the multiple integral in (5). What remains is that ρ(*r*) *is simple the electronic density (of the whole concerned system) in „***r***”* space point. Such simplified interpretation, apparently classics, preserves its quantum roots through the averaging (integral) over the many-electronic eigenfunction Ψ(**r**_1_,...,**r***_N_*) in (5). Alternatively, the explicit non-dependency of density on the wave function is also possible within the quantum statistical approach where the relation with partition function of the system (the global measure of the distribution of energetic states of a system) is mainly considered.

The major consequence of this theorem consists in defining of the total energy of a system as a function of the electronic density function in what is known as the density functional [8, 9, 12]:

(6) $$E[\rho ]=F_{HK}[\rho ]+C_{A}[\rho ],$$

from where the name of the theory. The terms of energy decomposition in (6) are identified as: the Hohenberg-Kohn density functional

(7) $$F_{HK}[\rho ]=T[\rho ]+V_{ee}[\rho ]$$

viewed as the summed electronic kinetic *T*[ρ] and electronic repulsion *V_ee_* [ρ], and the so called *chemical action* term [12–14]:

(8) $$C_{A}[\rho ]=\int \rho (\text{r})V(\text{r})d\text{r},$$

being the only explicit functional of total energy.

Although not entirely known the HK functional has a remarkably property: it is universally, in a sense that both the kinetic and inter-electronic repulsion are independent of the concerned system. The consequence of such universal nature offers the possibility that once it is exactly or approximately knew the HK functional *for a given external potential V*(**r**) remain valuable for any other type of potential *V’*(**r**) applied on the concerned many-electronic system. Let’s note the fact that *V*(**r**) *should be not reduced only to the Coulombic type* of potentials but is carrying the role of the generic potential applied, that could beg of either an electric, magnetic, nuclear, or even electronic nature as far it is external to the system fixed by the *N* electrons in the investigated system.

Once “in game” the external applied potential provides the second Hohenberg-Kohn (HK2) theorem. In short, HK2 theorem says that “the external applied potential is determined up to an additive constant by the electronic density of the *N*-electronic system ground state”.

In mathematical terms, the theorem assures the validity of the variational principle applied to the density functional (6) relation, i.e. [6]

(9) $$E[\bar{\rho }]\geq E[\rho ]\Leftrightarrow \delta E[\rho ]=0$$

for every electronic test density ρ̄ around the real density ρ of the ground state.

The proof of variational principle in (9), or, in other words, the one-to-one correspondence between the applied potential and the ground state electronic density, employs the *reduction ad absurdum* procedure. That is to assume that the ground state electronic density ρ(**r**) corresponds to two external potentials (*V*_1_, *V*_2_) fixing two associate Hamiltonians (*H*_1_, *H*_2_) to which two eigen-total energy (*E*_1_, *E*_2_) and two eigen-wave functions (Ψ_1_, Ψ_2_) are allowed. Now, if eigen-function Ψ_1_ is considered as the true one for the ground state the variational principle (9) will cast as the inequality:

(10) $$E_{1}[\rho ]=\int {\text{Ψ}}_{1}^{*}{\hat{H}}_{1}{\text{Ψ}}_{1}d\tau <\int {\text{Ψ}}_{2}^{*}{\hat{H}}_{1}{\text{Ψ}}_{2}d\tau =\int {\text{Ψ}}_{2}^{*}\left[{\hat{H}}_{2}+\left({\hat{H}}_{1}-{\hat{H}}_{2}\right)\right]{\text{Ψ}}_{2}d\tau$$

which is further reduced, on universality reasons of the HK functional in (6), to the form:

(11) $$E_{1}[\rho ]<E_{2}[\rho ]+\int \rho (\text{r})[V_{1}(\text{r})-V_{2}(\text{r})]d\text{r}\text{.}$$

On another way, if the eigen-function Ψ_2_ is assumed as being the one true ground state wavefunction, the analogue inequality springs out as:

(12) $$E_{2}[\rho ]<E_{1}[\rho ]+\int \rho (\text{r})[V_{2}(\text{r})-V_{1}(\text{r})]d\text{r.}$$

Taken together relations (11) and (12) generate, by direct summation, the evidence of the contradiction [2]:

(13) $$E_{1}[\rho ]+E_{2}[\rho ]<E_{1}[\rho ]+E_{2}[\rho ].$$

The removal of such contradiction could be done in a single way, namely, by abolishing, in a reverse phenomenologically order, the fact that two eigen-functions, two Hamiltonians and respectively, two external potential exist for characterizing the same ground state of a given electronic system. With this statement the HK2 theorem is formally proofed.

Yet, there appears the so called *V-representability* problem signaling the impossibility of an *a priori* selection of the external potentials types that are in bi-univocal relation with ground state of an electronic system [15–18]. The problem was revealed as very difficult at mathematical level due to the equivocal potential intrinsic behavior that is neither of universal nor of referential independent value. Fortunately, such principial limitation does not affect the general validity of the variational principle (9) regarding the selection of the energy of ground state level from a collection of states with different associated external potentials.

That because, the problem of *V*-representability can be circumvented by the so called *N-contingency features of ground state electronic density* assuring that, aside of the *N – integrability condition* (4), the candidate ground state densities should fulfill the *positivity condition* (an electronic density could not be negative) [17, 18]:

(14) ρ(r)≥0,  ∀|r|∈ℜ,

as well as the *non-divergent integrability condition* on the real domain (in relation with the fact that the kinetic energy of an electronic system could not be infinite – since the light velocity restriction):

(15) $$\int_{ℜ}{\left|\nabla \rho {\left(\text{r}\right)}^{1/2}\right|}^{2}d\text{r}<\infty \text{.}$$

Both (14) and (15) conditions are easy accomplished by every reasonable density, allowing the employment of the variational principle (9) in two steps, according to the so called *Levy-Lieb double minimization algorithm* [19]: one regarding the intrinsic minimization procedure of the energetic terms respecting all possible eigen-functions folding a trial electronic density followed by the external minimization over all possible trial electronic densities yielding the correct ground state (GS) energy density functional

(16) $$\begin{array}{l} E_{GS}=\underset{\rho }{\min}\left[\underset{\text{Ψ}\to \rho }{\min}\left(\int {\text{Ψ}}^{*}(T+V_{ee}+V)\text{Ψ}d\tau \right)\right]\\ =\underset{\rho }{\min}\left[\underset{\text{Ψ}\to \rho }{\min}\left(\int {\text{Ψ}}^{*}(T+V_{ee})\text{Ψ}d\tau \right)+\int \rho (\text{r})V(\text{r})d\text{r}\right]\\ \;\;\underset{\rho }{\min}(F_{HK}[\rho ]+C_{A}[\rho ])=\underset{\rho }{\min}(E[\rho ]). \end{array}$$

One of the most important consequences of the HK2 conveys the rewriting of the variational principle (9) in the light of above *N*-contingency conditions of the trial densities as the working Euler type equation:

(17) δ{E[ρ]−μN[ρ]}=0

from where, there follows the Lagrange multiplication factor with the functional definition:

(18) $$\mu ={\left(\frac{\delta E[\rho ]}{\delta \rho }\right)}_{\rho =\rho (V)}$$

this way introducing the *chemical potential* as the fundamental quantity of the theory. At this point, the whole chemistry can spring out since identifying the electronic systems electronegativity with the negative of the density functional chemical potential [9]:

(19) χ=−μ.

Up to now, the Hohenberg-Kohn theorems give new conceptual quantum tools for physico-chemical characterization of an electronic sample by means of electronic density and its functionals, the total energy and chemical potential (electronegativity). These positive density functional premises are in next analyzed towards elucidating of the quantum nature of the chemical bond and reactivity [20].

### 2.2. Optimized energy-electronegativity connection

Back from Paris, in the winter of 1964, Kohn met at the San Diego University of California his new post-doc Lu J. Sham with who propose to extract from HK1 & 2 theorems the equation of total energy of the ground state. In fact, they propose themselves to find the correspondent of the stationary eigen-equation of Schrödinger type, employing the relationship between the electronic density and the wave function.

Their basic idea consists in assuming a so called orbital basic set for the *N*-electronic system by replacing the integration in the relation (5) with summation over the virtual uni-electronic orbitals $\phi_{i},i=\bar{1,N}$, in accordance with Pauli principle, assuring therefore the HK1 frame with maximal spin/orbital occupancy [21]:

(20) $$\rho (\text{r})=\overset{N}{\underset{i}{\Sigma }}n_{i}|\varphi_{i}({\text{r})|}^{2},\;0\leq n_{i}\leq 1,\underset{i}{\Sigma }n_{i}=N.$$

Then, the *trial* total eigen-energy may be rewritten as density functional of eq. (6) nature expanded in the original form [22, 23]:

(21) $$\begin{array}{l} E[\rho ]=F_{HK}[\rho ]+C_{A}[\rho ]\\ =T[\rho ]+V_{ee}[\rho ]+C_{A}[\rho ]\\ =T_{s}[\rho ]+j[\rho ]+\left\{\left(T[\rho ]-T_{s}[\rho ])+(V_{ee}[\rho ]-J[\rho ]\right)\right\}+C_{A}[\rho ]\\ =\overset{N}{\underset{i}{\Sigma }}\int n_{i}\varphi_{i}^{*}(\text{r})\left[-\frac{1}{2}\nabla^{2}\right]\varphi_{i}(\text{r})d\text{r}+\frac{1}{2}\int \int \frac{\rho ({\text{r}}_{1})\rho ({\text{r}}_{2})}{r_{12}}d{\text{r}}_{1}d{\text{r}}_{2}+E_{xc}[\rho ]+\int V(\text{r})\rho (\text{r})d\text{r} \end{array}$$

where, the contribution of the referential uniform kinetic energy contribution

(22) $$T_{s}[\rho ]=\overset{N}{\underset{i}{\Sigma }}\int n_{i}\varphi_{i}^{*}(\text{r})\left[-\frac{1}{2}\nabla^{2}\right]\varphi_{i}(\text{r})d\text{r},$$

with the inferior index „s” referring to the „spherical” or homogeneous attribute together with the classical energy of Coulombic inter-electronic repulsion

(23) $$J[\rho ]=\frac{1}{2}\int \int \frac{\rho ({\text{r}}_{1})\rho ({\text{r}}_{2})}{r_{12}}d{\text{r}}_{1}d{\text{r}}_{2}$$

were used as the analytical vehicles to elegantly produce the exchange-correlation energy *E_xc_* containing exchange (*V_ee_*[ρ] – *J*[ρ]) and correlation (*T*[ρ] – *T_s_* [ρ]) heuristically introduced terms as the quantum effects of spin anti-symmetry over the classical interelectronic potential and of corrected homogeneous electronic movement, respectively.

Next, the trial density functional energy (21) will be optimized in the light of variational principle (17) as prescribed by the HK2 theorem. The combined result of the HK theorems will eventually furnish the new quantum energy expression of multi-electronic systems beyond the exponential wall of the wave function.

An instructive method for deriving such equation assume the same types of orbitals for the density expansion (20),

(24) $$\rho (\text{r})=N\varphi^{*}(\text{r})\varphi (\text{r})$$

that, without diminishing the general validity of the results, since preserving the *N*-electronic character of the system, highly simplifies the analytical discourse.

Actually, with the trial density (24) replaced throughout the energy expression in (21) has to undergo the minimization procedure (17) with the practical equivalent integral variant:

(25) $$\int \frac{\delta \left(E[\rho ]-\mu N[\rho ]\right)}{\delta \varphi^{*}}\delta \varphi^{*}d\text{r}=0\text{.}$$

Note that, in fact, we chose the variation in the conjugated uni-orbital φ^*^(**r**) in (25) providing from (24) the useful differential link:

(26) $$\delta \rho (\text{r})=N\varphi (\text{r})\delta \varphi^{*}(\text{r})\text{.}$$

Now, unfolding the equation (25) with the help of relations (21) and (24), together with fundamental density functional prescription (4), one firstly gets [24]:

(27) $$\frac{\delta }{\delta \varphi^{*}(\text{r})}\{-\frac{N}{2}\int \varphi^{*}(\text{r})\nabla^{2}\varphi (\text{r})d\text{r}+J[\rho ]+E_{xc}[\rho ]+N\int V(\text{r})\varphi^{*}(\text{r})\varphi (\text{r})d\text{r}-\mu N\int \varphi^{*}(\text{r})\varphi (\text{r})d\text{r}\}=0$$

By performing the required partial functional derivations respecting the uni-orbital φ^*^(**r**) and by taking account of the equivalence (26) in derivatives relating *J*[ρ] and *E_xc_* [ρ] terms, equation (27) takes the further form:

(28) $$-\frac{N}{2}\nabla^{2}\varphi (\text{r})+N\varphi (\text{r})\frac{\delta J[\rho ]}{\delta \rho }+N\varphi (\text{r})\frac{\delta E_{xc}}{\delta \rho }+NV(\text{r})\varphi (\text{r})-\mu N\varphi (\text{r})=0.$$

After immediate suppressing of the *N* factor in all the terms and by considering the exchange-correlation potential with the formal definition [4, 9]:

(29) $$V_{xc}(\text{r})={\left(\frac{\delta E_{xc}[\rho ]}{\delta \rho (r)}\right)}_{V(\text{r})},$$

equation (28) simplifies as [25, 26]:

(30) $$\left[-\frac{1}{2}\nabla^{2}+\left(V(\text{r})+\int \frac{\rho {(\text{r}}_{2})}{\left|\text{r}-{\text{r}}_{2}\right|}d{\text{r}}_{2}+V_{xc}(\text{r})\right)\right]\varphi (\text{r})=\mu \varphi (\text{r})\text{.}$$

Moreover, once introducing the so called *effective potential*:

(31) $$V_{eff}(\text{r})=V(\text{r})+\int \frac{\rho {(\text{r}}_{2})}{\left|\text{r}-{\text{r}}_{2}\right|}d{\text{r}}_{2}+V_{xc}(\text{r})$$

the resulted equation recovers the traditional Schrödinger shape:

(32) $$\left[-\frac{1}{2}\nabla^{2}+V_{eff}\right]\varphi (\text{r})=\mu \varphi (\text{r})\text{.}$$

The result (32) is fundamental and equally subtle. Firstly, it was proved that the joined Hohenberg-Kohn theorems are compatible with consecrated quantum mechanical postulates, however, still offering a generalized view of the quantum nature of electronic structures, albeit the electronic density was assumed as the foreground reality. In these conditions, the meaning of functions φ(**r**) is now unambiguously producing the analytical passage from configuration (3*N*-D) to real (3D) space for the whole system under consideration. Nevertheless, the debate may still remain because once equation (32) is solved the basic functions φ(**r**) generating the electronic density (24) and not necessarily the eigen-functions of the original system due to the practical approximations of the exchange and correlation terms appearing in the effective potential (31). This is why the functions φ(**r**) are used to be called as *Kohn-Sham (KS) orbitals*; they provide the orbital set solutions of the associate KS equations [3]:

(33) $$\left[-\frac{1}{2}\nabla^{2}+V_{eff}\right]\varphi_{i}(\text{r})=\mu_{i}\varphi_{i}(\text{r}),\;i=\bar{1,N}$$

once one reconsiders electronic density (24) back with general case (20).

Yet, equations (33), apart of delivering the KS wavefunctions φ*_i_*(**r**), associate with another famous physico-chemical figure, the orbital chemical potential μ*_i_*, which in any moment can be seen as the negative of the orbital electronegativities on the base of the relation (19).

Going now to a summative characterization of the above optimization procedure worth observing that the *N*-electronic in an arbitrary external *V*-potential problem is conceptual-computationally solved by means of the following self-consistent algorithm:

- It starts with a trial electronic density (20) satisfying the *N*-contingency conditions (14) and (15);
- With trial density the effective potential (31) containing exchange and correlation is calculated;
- With computed *V_eff_* the equations (33) are solved for $\phi_{i}(\text{r}),i=\bar{1,N}$;
- With the set of functions ${\left\{\phi_{i}(\text{r})\right\}}_{i=\bar{1,N}}$ the new density (20) is recalculated;
- The procedure is repeated until the difference between two consecutive densities approaches zero;
- Once the last condition is achieved one retains the last set ${\left\{\phi_{i}(\text{r}),\mu_{i}=-\chi_{i}\right\}}_{i=\bar{1,N}}$;
- The electronegativity orbital observed contributions are summed up from (33) with the expression:
(34) $$-\overset{N}{\underset{i}{\Sigma }}〈\chi_{i}〉=\overset{N}{\underset{i}{\Sigma }}\int n_{i}\varphi_{i}^{*}(\text{r})\left[-\frac{1}{2}\nabla^{2}+V_{eff}(\text{r})\right]\varphi_{i}(\text{r})d\text{r}=T_{s}[\rho ]+\int V_{eff}\rho (\text{r})d\text{r};$$
- Replacing in (34) the uniform kinetic energy, *T_s_* [ρ] from the general relation (21) the density functional of the total energy for the *N*-electronic system will take the final figure [9, 24]:
(35) $$E[\rho ]=-\overset{N}{\underset{i}{\Sigma }}〈\chi_{i}〉-\frac{1}{2}\int \int \frac{\rho ({\text{r}}_{1})\rho ({\text{r}}_{2})}{\left|{\text{r}}_{12}\right|}d{\text{r}}_{1}d{\text{r}}_{2}+\{E_{xc}[\rho ]-\int V_{xc}(\text{r})\rho (\text{r})d\text{r}\}.$$

showing that the optimized many-electronic ground state energy is directly related with global or summed over observed or averaged or expected orbital electronegativities. One can observe from (35) that even in the most optimistic case when the last two terms are hopefully canceling each other there still remains a (classical) correction to be added on global electronegativity in total energy. Or, in other terms, electronegativity alone is not enough to better describe the total energy of a many-electronic system, while its correction can be modeled in a global (almost classical) way. Such considerations stressed upon the accepted semiclassical behavior of the chemical systems, at the edge between the full quantum and classical treatments.

However, analytical expressing the total energy requires the use of suitable approximations, whereas for chemical interpretation of bonding the electronic localization information extracted from energy is compulsory. This subject is in next focused followed by a review of the popular energetic density functionals and approximations.

## 3. Electronic Localization Problem

### 3.1. From global functional to localization function. Localization in solids

The application of Hohenberg-Kohn theorems consecrate the crucial contributions of the so called “spherical” or homogeneous kinetic and of the exchange-correlation energy terms in a multi-electronic system’s ground state. However, the spherical electronic case corresponds to the non perturbed electronic system for which the Thomas-Fermi (TF) model was already advanced throughout totally ignoring exchange- correlation terms from the total energy shape:

(36) $$E_{TF}[\rho ]=T_{TF}[\rho ]+J[\rho ]+C_{A}[\rho ].$$

Such a referential picture is most useful in establishing the uniform electronic distribution by indicating the occupation of the all-possible electronic levels in a semiclassical quantum frame (without explicit exchange-correlation involvement). Actually, the Fermi sphere in a momentum space finely defines the total homogeneous kinetic energy as:

(37) $$\tau_{s}(\text{r})=\frac{p_{F}^{2}}{2m_{0}},$$

while the quantum nature of the kinetic energy (37) is covered by involving the quantum (Heisenberg) uncertainty

(38) $$\text{Δ}p_{x}\text{Δ}p_{y}\text{Δ}p_{z}\text{Δ}x\text{Δ}y\text{Δ}z≅h^{3}$$

in uniform density computation. This suggests that the density of states in the Fermi volume of the impulse *p_F_* has to be normalized to the inverse of the cube power of Planck constant *h*, while the density of electrons is reached by multiplying the density of states with the electron multiplicity 2(1/2)+1=2 for every occupied state. The obtained density-Fermi impulse relationship:

(39) $$\rho (\text{r})d\text{r}=2\frac{4}{3}\pi \frac{p_{F}^{3}}{h^{3}}d\text{r}\Rightarrow p_{F}(\text{r})={\left(\frac{3}{8\pi }\right)}^{1/3}h\rho {\left(\text{r}\right)}^{1/3}$$

allows the Thomas-Fermi kinetic energy unfolding as the density functional [9, 27–31]:

(40) $$T_{TF}[\rho ]=\frac{3}{5}\int \rho (\text{r})\tau_{s}(\text{r})d\text{r}=C_{TF}\int \rho {\left(\text{r}\right)}^{5/3}d\text{r},\;C_{TF}=\frac{3h^{2}}{10m_{0}}{\left(\frac{3}{8\pi }\right)}^{2/3}≅2.871[a.u.]$$

with the help of which the total Thomas-Fermi energy functional takes the form:

(41) $$E_{TF}[\rho ]=C_{TF}\int \rho {\left(\text{r}\right)}^{5/3}d\text{r}+\frac{1}{2}\iint \frac{\rho ({\text{r}}_{1})\rho ({\text{r}}_{2})}{\left|{\text{r}}_{12}\right|}d{\text{r}}_{1}d{\text{r}}_{2}+\int V(\text{r})\rho (\text{r})d\text{r}$$

that can be seen as the first approximation for the density functional total energy (21).

From physical point of view worth noted that the kinetic TF energy exactly corresponds to the total energy of the *free* electrons in a crystal, *V*(**r**)=0 in (41), equivalently with the fact that the electrons are not “feeling” the nuclei, i.e. electrostatic attractions are excluded, being as close each other to avoid reciprocal repelling. Such picture suggests that free electrons are completely non-localized leaving with the condition of complete cancellation of the electronic inter-repulsion; this feature may be putted formally as [32]:

(42) $$\frac{e^{2}}{\left|{\text{r}}_{1}-{\text{r}}_{2}\right|}\to \frac{\lambda e^{2}}{\left|{\text{r}}_{1}-{\text{r}}_{2}\right|},\lambda =0.$$

However, the model in which the (valence) electrons are completely free and are neither “feeling” the attraction nor the repulsion is certain not properly describing the nature of the chemical bond. In fact, this limitation was also the main objection brought to Thomas-Fermi model and to the atomic or molecular approximation of the homogeneous electronic gas or jellium model in solids. Nevertheless, the lesson is well served because Thomas-Fermi description may be regarded as the “inferior” extreme in quantum known structures while further exchange-correlation effects may be added in a perturbative manner.

The idea of introducing exchange and correlation effects as a perturbation of the homogeneous electronic system could be considered from the interpolation of the energetic terms for 0≤λ≤1 in (41). Parameter λ is defined as a *parameter of the electronic coupling*, with a slightly (adiabatically) scaling of the perturbation from the homogeneous electronic systems, λ=0, until the maximal inter-electronic interaction, λ=1 (in accordance with Pauli principle). Therefore, the overall interpolation $\int_{0}^{1}[•]d\lambda$ will be spread over the terms which contain the intermediate degree of exchange and correlation interactions; since it accounts for the electronic inter-repulsion while indexing the electronic presence/absence in a given spatial region the degree of *electronic localization* is in this way furnishes.

The coupling parameter λ will serve as a switcher between the referential Thomas-Fermi uniform case and the full interaction through the density limit:

(43) $$\underset{\lambda \to 1}{\lim}\rho_{\lambda }(\text{r})=\rho \left(\text{r}={\text{r}}_{1}\cap {\text{r}}_{2}\right).$$

Actually, the density (43) has a major role in defining exchange-correlation functionals. To see that let’s firstly consider the conditional electronic density *g*(**r**_1_, **r**_2_;λ) indicating that the electronic density in **r**_1_ is conditioned by the presence (localization) of another electron (any from the total *N* in the system) in **r**_2_. Mathematically, this is expressed by using the conditional probabilities:

(44) $$g({\text{r}}_{\text{1}},{\text{r}}_{\text{2}};\lambda )=\frac{\rho {(\text{r}=\text{r}}_{\text{1}}\cap {\text{r}}_{\text{2}})}{\rho ({\text{r}}_{\text{2}})}$$

fulfilling the Pauli principle by means of the integration rule:

(45) $$\int \rho \left({\text{r}}_{2}\right)g\left({\text{r}}_{1},{\text{r}}_{2};\lambda \right)d{\text{r}}_{2}=\int \rho \left({\text{r}}_{1}\cap {\text{r}}_{2}\right)d{\text{r}}_{2}=0$$

saying that the spatial average of the electronic reciprocal constraint vanishes. This behavior opens the possibility in introducing the conditional probability of electronic holes,

(46) $$h({\text{r}}_{1},{\text{r}}_{2};\lambda )=g({\text{r}}_{1},{\text{r}}_{2};\lambda )-1,$$

providing the associate integration rule [33]:

(47) $$\int \rho ({\text{r}}_{2})h({\text{r}}_{1},{\text{r}}_{2};\lambda )d{\text{r}}_{2}=-1$$

consecrating a sort of negative normalization of the exchange and correlation density of holes:

(48) $$\rho_{xc}({\text{r}}_{1},{\text{r}}_{2};\lambda )=\rho ({\text{r}}_{2})h({\text{r}}_{1},{\text{r}}_{2};\lambda ).$$

Now, once this exchange-correlation hole density is mediated over the coupling factor λ the averaged exchange-correlation density of holes is generated:

(49) $$\bar{\rho_{xc}}({\text{r}}_{1},{\text{r}}_{2})=\int_{0}^{1}\rho_{xc}({\text{r}}_{1},{\text{r}}_{2};\lambda )d\lambda$$

allowing the formal writing of exchange-correlation density functional from (29) as a generalized version of the inter-electronic interaction term (23):

(50) $$E_{xc}[\rho ]=\frac{1}{2}\iint \frac{\rho ({\text{r}}_{1})\bar{\rho_{xc}}({\text{r}}_{1},{\text{r}}_{2})}{r_{12}}d{\text{r}}_{1}d{\text{r}}_{2}=-\frac{1}{2}\int \frac{\rho ({\text{r}}_{1})}{\bar{R_{xc}}({\text{r}}_{1},\rho (\text{r}))}d{\text{r}}_{1}$$

with the help of the introduced radius of the λ*-mediated exchange-correlation density of holes*:

(51) $${\bar{R_{xc}}}^{-1}({\text{r}}_{1},\rho (\text{r})):=-\int \frac{\bar{\rho_{xc}}({\text{r}}_{1},{\text{r}}_{2},\rho \left(\text{r})\right)}{r_{12}}d{\text{r}}_{2}$$

The radius $\bar{R_{xc}}(\text{r})$ could be considered as a functional of density (43) with the leading term being defined in *the short limit of the distance*, i.e. being of the inter-particle average radius order,

(52) $$\frac{4\pi }{3}r_{0}^{3}=\frac{1}{\rho (\text{r})}\Rightarrow r_{0}={\left(\frac{3}{4\pi }\right)}^{1/3}\rho {\left(\text{r}\right)}^{-1/3},$$

known as the Wigner radius for indexing the volume of a sphere containing (*localizing*) a single electron (from the total of *N*) belonging to the density family (43), although, also other quantities accounting for electron localization such as the domain averaged Fermi hole of Ponec [34a] or the electron sharing index (also known as delocalization index) [34b] have been recently proposed (see also the forthcoming discussion).

In these conditions, the inverse radius (51) could be expressed around the inverse of the Wigner radius in a gradient density expansion [32]:

(53) $${\bar{R_{xc}}}^{-1}(\text{r},\rho (\text{r}))=F_{0}[\rho (\text{r})]+F_{21}[\rho (\text{r})]\nabla^{2}\rho (\text{r})+F_{22}[\rho (\text{r})]\sum_{i=1}^{N}\left(\nabla_{i}\rho (\text{r})\right)\left(\nabla_{i}\rho (\text{r})\right)+...$$

while, by considering it back in exchange-correlation energy (50) produces, after the integration by parts, the generalized gradient density functional:

(54) $$E_{xc}=\int G_{0}[\rho (\text{r})]\rho (\text{r})d\text{r}+\int G_{2}[\rho (\text{r})]{\left(\nabla \rho (\text{r})\right)}^{2}d\text{r}+\int \left[G_{4}[\rho (\text{r})]{\left(\nabla^{2}\rho (\text{r})\right)}^{2}+...\right]d\text{r}+...$$

The restriction to the first term of the series (54) corresponds to the cases where the spatial distance of variation in electronic density highly exceeds the corresponding Wigner radius (52) this way producing the famous *local density approximation* (LDA) [34c]:

(55) $$E_{xc}^{LDA}[\rho ]=\int e_{xc}[\rho (\text{r})]\rho (\text{r})d\text{r}$$

with *e_xc_* being the exchange and correlation density per particle, that can be further approximated (see bellow) as [35–40]:

(56) $$e_{xc}[\rho ]=e_{x}[\rho ]+e_{c}[\rho ]=\left(-\frac{0.458}{r_{0}}\right)+\left(-\frac{0.44}{r_{0}+7.8}\right)[a.u.].$$

In fact, the LDA stands as the immediate step after TF approximation; it can be extended also for systems with un-pair spins by the so called *local spin density approximation* (LSDA) [41–50]:

(57) $$E_{xc}[\rho =\rho_{↑}+\rho_{↓}]=E_{xc}[\rho_{↑}]+E_{xc}[\rho_{↓}],$$

while further inclusion of the gradient terms in (54) establishes the *general gradient approximation* (GGA).

Worth noting that when undertaken GGA, beside the gradient terms arising in exchange-correlation energy, the gradient correction of the kinetic energy functional has to be as well considered providing terms of which the standard one takes the von Weizsäcker form [51, 52]:

(58) $$\tau_{W}(\text{r})=\frac{1}{8}\frac{{\left|\nabla \rho (\text{r})\right|}^{2}}{\rho (\text{r})}.$$

While more analytical discussions about various approximations and density functionals are bellow presented in a separate chapter, here we would like only to present the practical difference between the local and gradient density approximations for a solid state case.

**Figure 1.**
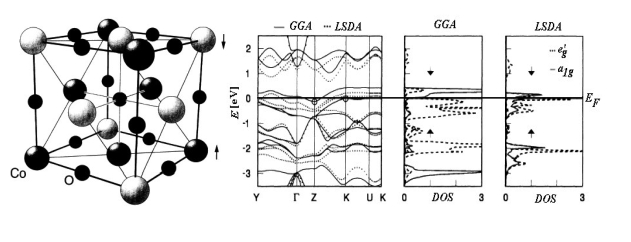
Left: the anti-ferromagnetic structure CoO; right: the band structure and the density of state (DOS) in LSDA and GGA approximations, respectively [53]. The upper and down arrows are associated with the spin orbital projections.

For instance, Figure 1 presents the band structure and the density of states (DOS) for the *R* $\bar{3}$*m* oxide of the Cobalt transitional metal (CoO) calculated with either LSDA and GGA approximations [53].

Regarding the energy bands there can be noted that, around the Fermi level *E_F_*, LSDA approach is less relevant in indicating the energetically gap respecting the GGA computation. The difference is even more drastic in DOS representations for employed approximations in *d* orbital separation (t_2g_=a_1g_+e_g_’) due to the central ion of cobalt trigonal symmetry coordination. In fact, with LSDA a strong mixing of the orbitals a_1g_ and e_g_’ is recorded while in the case of GGA-DOS the bands with the symmetry a1g are up and down shifted for the respective down and up spin projections resulting a clear separation from states with e_g_’ symmetry.

Nonetheless, at the level of bands structure of the solids and crystals an inevitable *localization paradox* emerges namely, to use the real 3D electronic densities in furnish a localization description in the reciprocal (energy) space.

Recently, it was found a way to avoid the electronic localization paradox through introducing specific electronic localization functions (ELF) in real space. Nevertheless, an ELF should relay on combination of the gradient and homogeneous energetic density functionals, in accordance with Pauli principle, shaping for instance as [54]:

(59) $$ELF={\left(1+{\left[\frac{\tau_{s}(\text{r})-\tau_{W}(\text{r})}{T_{TF}[\rho (\text{r})]}\right]}^{2}\right)}^{-1}$$

by emphasizing the excess of the kinetic energy difference τ_s_(**r**)- τ*_W_*(**r**) „normalized” to the referential kinetic TF homogeneous behavior.

**Figure 2.**
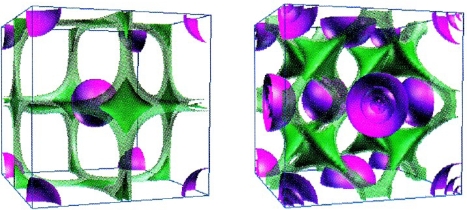
The localization domains for Li (left) and Sc (right) crystals based on the electronic localization function ELF (59) [55].

Worth remarking that the localization function (59) acts like a sort of density, with values between 0 and 1 corresponding with maximum delocalization and localization, respectively. This heuristically proposal has the merit to give an analytical reflection of the qualitative *valence shell electron pair repulsion* (VSEPR) geometric model [56], with the immediate consequence in topological characterization of the chemical bond [57]. In solid state case, the reliability of above ELF in describing the chemical bond in real space is illustrated in Figure 2 for the Li and Sc crystals. Atomic and molecular levels are in next section illustrated with which occasion further ELF characterization and developments are presented.

### 3.2. Localization in atoms and molecules

The definitions that are currently used in the classification of chemical bonds are often imprecise, as they are derived from approximate theories. Based on the topological analysis local, quantum-mechanical functions related to the Pauli Exclusion Principle may be formulated as “localization attractors” of bonding, non-bonding, and core types. Bonding attractors lie between nuclei core attractors and characterize shared electron interactions. The spatial arrangement of bond attractors allows for an absolute classification of ionic *versus* covalent bond to be derived from electronic density combined functions [58].

Most modern classifications of the chemical bond are based on Lewis’ theory and rely on molecular-orbital and valence-bond theories with schemes involving linear combination of atomic orbitals (LCAO). However, electron density alone does not easily reveal the consequences of the Pauli Exclusion Principle on bonding nature. While VSEPR theory indicates that the Pauli principle is important for understanding chemical structures, it has been reformulated in terms of maxima of electronic density’s Laplacian −∇^2^ρ(**r**) [56]. Next, the exchange-correlation density functional concept was employed to achieve the coordinate-space dynamical correlation in an inhomogeneous electron gas. This way the exchange-correlation energy (50) further re-expresses like [32, 54]

(60) $$E_{xc}=\frac{1}{2}\sum_{\alpha \alpha^{\prime }}\iint \frac{\rho ({\text{r}}_{1})\rho ({\text{r}}_{2})}{r_{12}}h_{XC}^{\alpha \alpha^{\prime }}d{\text{r}}_{1}d{\text{r}}_{2},$$

by accounting for the four α-spin types of interactions through the “hole” functions [40, 59]

(61) $$h^{\alpha \alpha^{\prime }}({\text{r}}_{1,}{\text{r}}_{2})=\frac{P_{2}^{\alpha \alpha^{\prime }}({\text{r}}_{1},{\text{r}}_{2})}{\rho_{\alpha }({\text{r}}_{1})}--\rho_{\alpha }({\text{r}}_{2})$$

where *P*_2_(**r**_1_,**r**_2_) stands for the two-body or pair probability density or correlation probability of the arbitrary electrons „1 and 2”, defined in terms of the *N*-body wave function Ψ as follows [9]:

(62) $$P_{2}({\text{r}}_{1,}{\text{r}}_{2})=N(N-1)\iint ...\int {\text{Ψ}}^{*}({\text{r}}_{1,}{\text{r}}_{2},{\text{r}}_{3,}...,{\text{r}}_{N})\text{Ψ}({\text{r}}_{1,}{\text{r}}_{2},{\text{r}}_{3,}...,{\text{r}}_{N})d{\text{r}}_{3}...d{\text{r}}_{N}\;\text{.}$$

Within the density functional theory the electrons of a pair of electrons or a bond can be considered as belonging to an inhomogeneous continuum gas. In analytical terms this was translated as the ELF (59) index as combining the homogeneous and inhomogeneous behaviors of a many-electronic-nuclei system.

Nevertheless, recently, the Markovian analytical shape of an ELF was shown to have the general qualitative form [60]:

(63) $$ELF=f^{-1}\left(\frac{g(\text{r})}{h(\text{r})}\right)$$

with the limiting constrains

(64) $$\lim ELF=\{\begin{array}{c} 0,\nabla \rho (\text{r})\;\gg \;\rho (\text{r})\\ 1,\nabla \rho (\text{r})\;\ll \;\rho (\text{r}) \end{array}$$

assuring the fulfillment of the Heisenberg and Pauli principles. To clarify this [60], we make recourse to the Heisenberg principle, comprised in ELF. When density gradient dominates,∇ρ≫ρ then *g≫h* in (63), and *f*(∞) should accounted for the infinite error in assigning of momentum, therefore indicating a precisely spatial localization of electrons; thus *f*(∞) = ∞ and ELF→0. In such, *the meaning of ELF is associated with the error in spatial localization of electrons*, being zero when the electrons are precisely located. On the contrary, when ρ≫∇ρ then *h≫g* in (63), and the resulting *f*(0) indicates the minimum error in defining of momentum and should provide the maximum uncertain of spatial distribution; in such *f*(0)=1 and ELF→1, where 1 stands here for 100% of coordinate localization error.

In this context, when the inverse of difference in local kinetic terms is involved, the ELF is interpreted as the *error in localization* of electrons within traps rather than where they have peaks of spatial density, as is frequently misinterpreted in literature [61], albeit recent extensions of ELF have used the correlated (Hartree-Fock) wavefunctions, through the conditional pair probability, however not using the “kinetic energy approach” [62–64].

Among various classes of Markovian ELFs the most representative and efficient one was proposed as having the form [60]:

(65) $$ELF=\exp\{-\frac{3}{2}{\left[\frac{g(\text{r})}{h(\text{r})}\right]}^{2}\}$$

with the components:

(66) $$g(\text{r})=\frac{1}{2}\sum_{i}{\left[\nabla \varphi_{i}(\text{r})\right]}^{2}-\frac{1}{8}\frac{{\left[\nabla \rho (\text{r})\right]}^{2}}{\rho (\text{r})},h(\text{r})=\frac{3}{10}{\left(3\pi^{2}\right)}^{2/3}{\left[\rho (\text{r})\right]}^{5/3}$$

being responsible for the gradient (*g*) and the homogenous (*h*) density distributions, respectively. In this frame, the *ELF* information prescribe that as it has values closer to zero as the better electronic localization is providing, according with the limits (64).

Going to a particular application of this scheme the atomic level is firstly presented for the special case of Li atom. The main stages consist in:

- Choosing the basis of the atomic functions [65]:
(67) $$\begin{array}{c} f_{1}^{Li}(r)=8.863248r\exp(-2.698r),\\ f_{2}^{Li}(r)=0.369721r^{5/2}\exp(-0.797r) \end{array}$$

such that to fulfill the natural (radial) normalization conditions

(68) $$\int_{0}^{\infty }{\left[f_{n}^{Li}(r)\right]}^{2}dr=1,n=1,2$$

- Generating the orthonormal orbital eigen-waves, here according with the Gram-Schmidt algorithm among shells and sub-shells:
(69) $$\begin{array}{c} \varphi_{1s}^{Li}(r)=f_{1}^{Li}(r),\;\varphi_{2p}^{Li}(r)=f_{2}^{Li}(r),\\ \varphi_{2s}^{Li}(r)=C_{2s}^{Li}\left[\varphi_{2p}^{Li}(r)-\alpha \varphi_{1s}^{Li}(r)\right]\\ =-1.2226\exp(-2.698r)+0.373222r^{5/2}\exp(-0.797r) \end{array}$$

ensuring the additionally constraints:

(70) $$\int_{0}^{\infty }\varphi_{2s}^{Li}(r)\varphi_{1s}^{Li}(r)dr=0,\;\int_{0}^{\infty }{\left[\varphi_{2s}^{Li}(r)\right]}^{2}d\text{r}=1\text{.}$$

- Generating the working overall electronic density
(71) $$\rho_{Li}(r)=2{\left[\varphi_{1s}^{Li}(r)\right]}^{2}+{\left[\varphi_{2s}^{Li}(r)\right]}^{2}$$

that satisfies the spatial (radial) global *N*-integral condition (4):

(72) $$\int_{0}^{\infty }\rho_{Li}(r)dr=3$$

**Figure 3.**
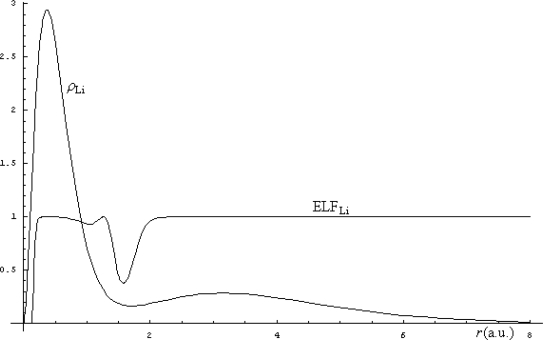
Comparison of the radial density given by (71) with the electron localization function (65) with components (66) for the simplified self-consistent approximation (67)–(70) for Li atomic structure [66].

The electronic density (71) is then used for computation of the Markovian ELF (65), while their comparison is in Figure 3 illustrated.

From the Figure 3, there appears that the smooth delocalization of electrons of Li represented by density structure is removed by the electronic localization function by clearly indicating where are the regions where the electronic realm is with less uncertainty detected. This way the ELF indicates merely where the electronic transitions behave like a step-function. In this respect, ELF can be regarded as the complement of electronic density being a better indicator of the regions where the bonding may arise. For instance, in the case of Li atomic structure, the fact that the ELF does not displays localization over the second shell (due to its values approaching unity in this range) indicates a natural tendency for releasing the outermost electron to the (virtual) neighborhood atoms with uncompleted last shell(s) while preserving its delocalization feature across the bond. As such the lithium hydride (LiH) bond is expected to be formed with a certain degree of ionicity in resonance with its covalence: LiH ↔ Li^+^H^−^.

**Figure 4.**
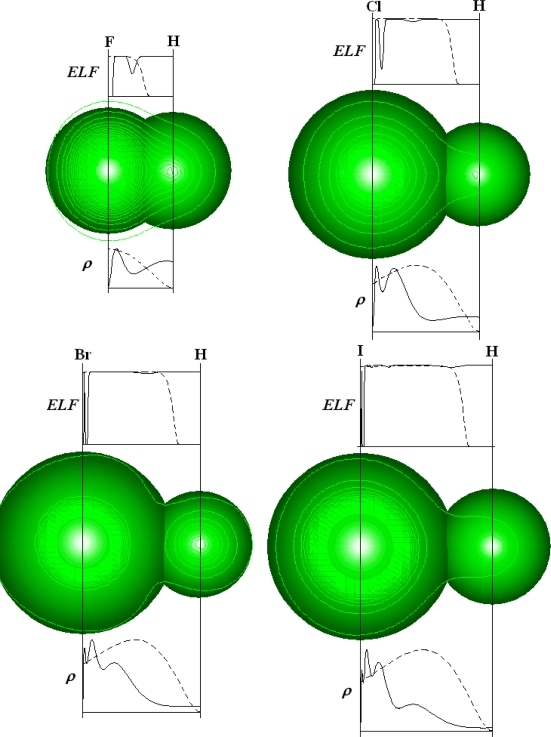
Comparative analysis of the charge density contours, electronic localization functions (ELFs), and radial densities for the H (dashed lines), F, Cl, Br, and I (full lines) atoms in molecular combinations HF, HCl, HBr, and HI, respectively [24].

The reliability of ELF to quantify the local tendency of atoms to form bonds and aggregates can be further exemplified to diatomic molecules, while the particular cases of HF, HCl, HBr and HI structures are considered in Figure 4. In the bonding region, i.e. in the space between the hydrogen and halogen atomic centers in H-X molecules there are represented both the electron densities, computed upon above recipe [65], and the associate Markovian ELFs for the concerned atoms-in-molecules (AIM).

Figure 4 clearly shows that while the crossing of hydrogen and halogen radial densities does not provide the right bonding region in HCl, HBr, and HI cases, the corresponding ELFs cross-lines of AIM finely indicate the frontier of atomic basins in hydracids thus confirming the ELF reliability in identifying chemical bonds and bonding.

One can equally say that in the crossing vicinity of *AIM-ELF*s the electrons are at the same time completely localized (for bonding with ELF_X_ – ELF_H_ →0) and completely delocalized for atomic systems (with ELF_X,H_ →1), according to above the *ELF* definition and present signification.

In other words it can be alleged that *ELF application on chemical bond helps in identifying the molecular region in which the electrons undergo the transition from the complete delocalization in atoms to complete localization in molecular bonding behavior*.

Actually, it also proves that localization issue of ionic and covalent classification of bonds may be solved by a “continuous” quantum reality. Such a feature gives, nevertheless, an in-depth understanding of the quantum nature of the chemical bond by associating the mysterious pairing of electrons issue to an analytical function able to distinguish the narrow regions of molecular space where the Heisenberg and Pauli principles are jointly satisfied through the ELF’s extreme values. Even more such sharp differentiation between 0 and 1 in atomic and molecular ELF values offers the future possibility in quantifying the chemical bond and bonding in the frame of quantum information theory [67].

## 4. Popular Energetic Density Functionals

Since the terms of total energy are involved in bonding and reactivity states of many-electronic systems, i.e. the kinetic energetic terms in ELF topological analysis or the exchange and correlation density functionals in chemical reactivity in relation with either localization and chemical potential or electronegativity, worth presenting various schemes of quantification and approximation of these functionals for better understanding their role in chemical structure and dynamics.

### 4.1. Density functionals of kinetic energy

When the electronic density is seen as the diagonal element ρ(**r_1_**) = ρ(**r**_1_,**r**_1_) the kinetic energy may be generally expressed from the Hartree-Fock model, through employing the single determinant ρ(**r**_1_,**r**′_1_), as the quantity [68]:

(73) T[ρ]=−12∫[∇r'12ρ(r1,r'1)]r1=r'1dr1;

it may eventually be further written by means of the thermodynamical (or statistical) density functional [69]:

(74) $$T_{\beta }=\frac{3}{2}\int \rho (\text{r})k_{B}T(\text{r})d\text{r}=\frac{3}{2}\int \rho (\text{r})\frac{1}{\beta (\text{r})}d\text{r}$$

that supports various specializations depending on the statistical factor particularization β.

For instance, in LDA approximation, the temperature at a point is assumed as a function of the density in that point, β(**r**) = β(ρ(**r**)); this may be easily reached out by employing the scaling transformation to be [70]

(75) $$\rho_{\lambda }(\text{r})=\lambda^{3}\rho (\lambda \text{r})\Rightarrow T[\rho_{\lambda }]=\lambda^{2}T[\rho ],\;\lambda =ct.,$$

providing that

(76) $$\beta (\text{r})=\frac{3}{2}C\rho^{-2/3}(\text{r}),$$

a result that helps in recovering the traditional (Thomas-Fermi) energetic kinetic density functional form

(77) $$T[\rho ]=C\int \rho^{5/3}(\text{r})d\text{r},$$

while the indeterminacy remained is smeared out in different approximation frames in which also the exchange energy is evaluated. Note that the kinetic energy is generally foreseen as having an intimate relation with the exchange energy since both are expressed in Hartree-Fock model as determinantal values of ρ(**r**_1_,**r**′_1_), see below.

Actually, the different LDA particular cases are derived by equating the total number of particle *N* with various realization of the integral

(78) N=12∫∫|ρ(r1,r'1)|2dr1dr1'

by rewritting it within the inter-particle coordinates frame:

(79) r=0.5(r1+r1'),s=r1−r1'

as:

(80) $$N=\frac{1}{2}\iint {\left|\rho \left(\text{r}+\text{s}/2,\text{r}-\text{s}/2\right)\right|}^{2}d\text{r}d\text{s}$$

followed by spherical averaged expression:

(81) $$N=2\pi \;\iint \rho^{2}(\text{r})\text{Γ}(\text{r},s)d\text{r}s^{2}ds$$

with

(82) $$\text{Γ}(\text{r},s)=1-\frac{s}{\beta (\text{r})}+\ldots$$

The option in choosing the Γ(**r**, *s*) series (82) so that to converge in the sense of charge particle integral (81) fixes the possible cases to be considered [68]:

1. the Gaussian resummation uses:
(83) $$\text{Γ}(\text{r},s)≅{\text{Γ}}_{G}(\text{r},s)=\text{exp}\left(-\frac{s^{2}}{\beta (\text{r})}\right)$$
2. the trigonometric (uniform gas) approximation looks like:
(84) $$\text{Γ}(\text{r},s)≅{\text{Γ}}_{T}(\text{r},s)=9\frac{{\left(\sin t-t\cos t\right)}^{2}}{t^{6}},t=s\sqrt{\frac{5}{\beta (\text{r})}}$$

In each of (83) and (84) cases the LDA-β function (76) is firstly replaced; then, the particle integral (81) is solved to give the constant *C* and then the respective kinetic energy density functional of (77) type is delivered; the results are [68]:

1. in Gaussian resummation:
(85) $$T_{G}^{LDA}=\frac{3\pi }{2^{5/3}}\int \rho^{5/3}(\text{r})d\text{r},$$
2. whereas in trigonometric approximation
(86) $$T_{TF}^{LDA}=\frac{3}{10}{\left(3\pi^{2}\right)}^{2/3}\int \rho^{5/3}(\text{r})d\text{r}$$

one recovers the Thomas-Fermi formula type that closely resembles the original TF (40) formulation.

In next one will consider the non-local functionals; this can be achieved through the gradient expansion in the case of slowly varying densities – that is assuming the expansion [52]:

(87) $$\begin{array}{c} T=\int d\text{r}\left[\tau (\rho_{↑})+\tau (\rho_{↓})\right]\;\;\\ \;=\int d\text{r}\overset{\infty }{\underset{m=0}{\Sigma }}\left[\tau_{2m}(\rho_{↑})+\tau_{2m}(\rho_{↓})\right]\\ \;=\int d\text{r}\overset{\infty }{\underset{m=0}{\Sigma }}\tau_{2m}(\rho )\\ \;=\int d\text{r}\tau (\rho )\;\;\; \end{array}$$

The first two terms of the series respectively covers: the Thomas Fermi typical functional for the homogeneous gas

(88) $$\tau_{0}(\rho )=\frac{3}{10}{\left(6\pi^{2}\right)}^{2/3}\rho^{5/3}$$

and the Weizsäcker related first gradient correction:

(89) $$\tau_{2}(\rho )=\frac{1}{9}\tau_{W}(\rho )=\frac{1}{72}\frac{{\left|\nabla \rho \right|}^{2}}{\rho }.$$

They both correctly behave in asymptotic limits:

(90) $$\tau (\rho )=\{\begin{array}{ll} \tau_{0}(\rho )=\tau_{2}(\rho ) & \ldots \nabla \rho \ll (\text{far}\;\text{from}\;\text{nucleus})\\ 9\tau_{2}(\rho )=\tau_{W}(\rho )=\frac{1}{8}\frac{{\left|\nabla \rho \right|}^{2}}{\rho } & \ldots \nabla \rho \gg (\text{close}\;\text{to}\;\text{nucleus}) \end{array}$$

However, an interesting resummation of the kinetic density functional gradient expansion series (87) may be formulated in terms of the Padé-approximant model [71]:

(91) $$\tau (\rho )=\tau_{o}(\rho )P_{4,3}(x)$$

with

(92) $$P_{4,3}(x)=\frac{1+0.95x+a_{2}x^{2}+a_{3}x^{3}+9b_{3}x^{4}}{1-0.05x+b_{2}x^{2}+b_{3}x^{3}}$$

and where the *x*-variable is given by

(93) $$x=\frac{\tau_{2}(\rho )}{\tau_{0}(\rho )}=\frac{5}{108}\frac{1}{{\left(6\pi^{2}\right)}^{2/3}}\frac{{\left|\nabla \rho \right|}^{2}}{\rho^{8/3}},$$

while the parameters *a*_2_, *a*_3_, *b*_2_, and *b*_3_ are determined by fitting them to reproduce Hartree-Fock kinetic energies of He, Ne, Ar, and Kr atoms, respectively [72]. Note that Padé function (92) may be regarded as a sort of generalized ELF susceptible to be further used in bonding characterizations.

### 4.2. Density functionals of exchange energy

Starting from the Hartree-Fock framework of exchange energy definition in terms of density matrix [73],

(94) K[ρ]=-14∬|ρ(r1,r'1)|2|r1−r'1|dr1dr1',

within the same consideration as before, we get that the spherical averaged exchange density functional

(95) $$K=\pi \iint \rho^{2}(\text{r})\text{Γ}(\text{r},s)d\text{r}sds$$

takes the particular forms [68]:

1. in Gaussian resummation:
(96) $$K_{G}^{LDA}=\frac{1}{2^{1/3}}\int \rho^{4/3}(\text{r})d\text{r};$$
2. and in trigonometric approximation (recovering the Dirac formula):
(97) $$K_{G}^{LDA}=-\frac{3}{4}{\left(\frac{3}{\pi }\right)}^{1/3}\int \rho^{4/3}(\text{r})d\text{r}.$$

Alternatively, by paralleling the kinetic density functional previous developments the gradient expansion for the exchange energy may be regarded as the density dependent series [74]:

(98) $$\begin{array}{c} K=\overset{\infty }{\underset{n=0}{\Sigma }}K_{2n}(\rho )\\ =\int d\text{r}\overset{\infty }{\underset{n=0}{\Sigma }}k_{2n}(\rho )\\ \;=\int d\text{r}k(\rho ) \end{array}$$

while the first term reproduces the Dirac LDA term [75, 76]:

(99) $$k_{0}(\rho )=-\frac{3}{2}{\left(\frac{3}{4\pi }\right)}^{1/3}\rho^{4/3}$$

and the second term contains the density gradient correction, with the Becke proposed approximation [77]:

(100) $$k_{2}(\rho )=-b\frac{\frac{{\left|\nabla \rho \right|}^{2}}{\rho^{4/3}}}{{\left(1+d\frac{{\left|\nabla \rho \right|}^{2}}{\rho^{8/3}}\right)}^{a}}$$

where the parameters *b* and *d* are determined by fitting the *k*_0_+*k*_2_ exchange energy to reproduce Hartree-Fock counterpart energy of He, Ne, Ar, and Kr atoms, and where for the *a* exponent either 1.0 or 4/5 value furnishes excellent results. However, worth noting that when analyzing the asymptotic exchange energy behavior, we get in small gradient limit [77]:

(101) $$k(\rho )\overset{\nabla \rho \ll }{\to }k_{0}(\rho )-\frac{7}{432\pi {\left(6\pi^{2}\right)}^{1/3}}\frac{{\left|\nabla \rho \right|}^{2}}{\rho^{4/3}}$$

whereas the adequate large-gradient limit is obtained by considering an arbitrary damping function as multiplying the short-range behavior of the exchange-hole density, with the result:

(102) $$k(\rho )\overset{\nabla \rho \gg }{\to }c\rho^{4/5}{\left|\nabla \rho \right|}^{2/5}$$

where the constant *c* depends of the damping function choice.

Next, the Padé-resummation model of the exchange energy prescribes the compact form [74]:

(103) $$k(\rho )=\frac{10}{9}\frac{k_{0}(\rho )}{P_{4,3}(x)}$$

with the same Padé-function (92) as previously involved when dealing with the kinetic functional resummation. Note that when *x*=0, one directly obtains the Ghosh-Parr functional [78]:

(104) $$k(\rho )=\frac{10}{9}k_{0}(\rho )\;\;.$$

Moreover, the asymptotic behavior of Padé exchange functional (103) leaves with the convergent limits:

(105) $$k(\rho )=\{\begin{array}{ll} \frac{10}{9}\left(k_{0}+\frac{15}{17}\frac{7}{432\pi {\left(6\pi^{2}\right)}^{1/3}}\frac{{\left|\nabla \rho \right|}^{2}}{\rho^{4/3}}\right) & \ldots \;\;\;x\to 0\;(\mathrm{SMALL}\;\mathrm{GRADIENTS})\;\\ -12\pi \frac{\rho^{2}}{{\left|\nabla \rho \right|}^{2}} & \ldots \;\;x\to \infty \;\;(\mathrm{LARGE}\;\mathrm{GRADIENTS}) \end{array}\;$$

Once again, note that when particularizing small or large gradients and fixing asymptotic long or short range behavior, we are recovering the various cases of bonding modeled by the electronic localization recipe as provided by ELF’s limits (64).

Another interesting approach of exchange energy in the gradient expansion framework was given by Bartolotti through the two-component density functional [79]:

(106) $$K[\rho ]=C(N)\int \rho {\left(\text{r}\right)}^{4/3}d\text{r}+D(N)\int {\text{r}}^{2}\frac{{\left|\nabla \rho \right|}^{2}}{\rho^{2/3}}dr,$$

where the *N*-dependency is assumed to behave like:

(107) $$C(N)=C_{1}+\frac{C_{2}}{N^{2/3}},\;\;D(N)=\frac{D_{2}}{N^{2/3}}$$

while the introduced parameters *C*_1_, *C*_2_, and *D*_2_ were fond with the exact values [80–82]:

(108) $$C_{1}=-\frac{3}{4}\pi^{1/3},\;\;C_{2}=-\frac{3}{4}\pi^{1/3}\left[1-{\left(\frac{3}{\pi^{2}}\right)}^{1/3}\right],\;\;D_{2}=\frac{\pi^{1/3}}{729}\;\;.$$

Worth observing that the exchange Bartolotti functional (106) has some important phenomenological features: it scales like potential energy, fulfills the non-locality behavior through the powers of the electron and powers of the gradient of the density, while the atomic cusp condition is preserved [83].

However, density functional exchange-energy approximation with correct asymptotic (long range) behavior, i.e. satisfying the limits for the density

(109) $$\underset{r\to \infty }{\lim}\;\rho_{\sigma }=\exp(-a_{\sigma }r)$$

and for the Coulomb potential of the exchange charge, or Fermi hole density at the reference point **r**

(110) $$\underset{r\to \infty }{\lim}U_{X}^{\sigma }=-\frac{1}{r},\;\;\sigma =\alpha (or↑),\;\beta (or↓)\ldots spin\;\;states$$

in the total exchange energy

(111) $$K[\rho ]=\frac{1}{2}\sum_{\sigma }\int \rho_{\sigma }U_{X}^{\sigma }d\text{r}\;,$$

was given by Becke *via* employing the so called semiempirical (SE) modified gradient-corrected functional [77]:

(112) $$K^{SE}=K_{0}-\beta \sum_{\sigma }\int \rho_{\sigma }^{4/3}\frac{x_{\sigma }^{2}(\text{r})}{1+\gamma x_{\sigma }^{2}(\text{r})}d\text{r}\;,\;\;K_{0}=\int d\text{r}k_{0}[\rho (\text{r})],\;\;x_{\sigma }(\text{r})=\frac{\left|\nabla \rho_{\sigma }(\text{r})\right|}{\rho_{\sigma }^{4/3}(\text{r})}$$

to the working single-parameter dependent one [84]:

(113) $$K^{B88}=K_{0}-\beta \sum_{\sigma }\int \rho_{\sigma }^{4/3}(\text{r})\frac{x_{\sigma }^{2}(\text{r})}{1+6\beta x_{\sigma }(\text{r})\sinh^{-1}x_{\sigma }(\text{r})}d\text{r}$$

where the value β = 00042[*a.u.*] was found as the best fit among the noble gases (He to Rn atoms) exchange energies; the constant *a*_σ_ is related to the ionization potential of the system.

Still, having different exchange approximation energetic functionals as possible worth explaining from where such ambiguity eventually comes. To clarify this, it helps in rewriting the starting exchange energy (94) under the formally exact form [85]:

(114) $$K[\rho ]=\sum_{\sigma }\int \rho_{\sigma }(\text{r})k[\rho_{\sigma }(\text{r})]g[x_{\sigma }(\text{r})]d\text{r}$$

Where the typical components are identified as:

(115) $$k[\rho ]=-A_{X}\rho^{1/3},\;\;A_{X}=\frac{3}{2}{\left(\frac{3}{4\pi }\right)}^{1/3},$$

while the gradient containing correction *g*(*x*) is to be determined.

Firstly, one can notice that a sufficiency condition for the two exchange integrals (111) and (114) to be equal is that their integrands, or the exchange potentials, to be equal; this provides the leading gradient correction:

(116) $$g_{0}(x)=\frac{1}{2}\frac{U_{X}(\text{r}(x))}{\;k[\rho (\text{r}(x))]}\;$$

with **r**(*x*) following from *x*(**r**) by (not unique) inversion.

Unfortunately, the above “integrity” condition for exchange integrals to be equal is not also necessary, since any additional gradient correction

(117) $$g(x)=g_{0}(x)+\Delta g(x)$$

fulfills the same constraint if it is chosen so that

(118) $$\int \rho^{4/3}(\text{r})\Delta g(x(\text{r}))d\text{r}=0$$

or, with the general form:

(119) $$\Delta g(x)=f(x)-\frac{\int \rho^{4/3}(\text{r})f(x(\text{r}))d\text{r}}{\int \rho^{4/3}(\text{r})d\text{r}}$$

being *f*(*x*) an arbitrary function.

Nonetheless, if, for instance, the function *f*(*x*) is specialized so that

(120) $$f(x)=-g_{0}(x)$$

the gradient correcting function (117) becomes:

(121) $$g(x)=-\frac{1}{2A_{X}}\frac{\int \rho (\text{r})U_{X}(\text{r})d\text{r}}{\int \rho^{4/3}(\text{r})d\text{r}}\equiv \alpha_{X}$$

recovering the Slater’s famous *X*_α_ method for exchange energy evaluations [86, 87]:

(122) $$K[\rho ]=-\alpha_{X}A_{x}\int \rho^{4/3}(\text{r})d\text{r}.$$

Nevertheless, the different values of the multiplication factor α*_X_* in (122) can explain the various forms of exchange energy coefficients and forms above. Moreover, following this conceptual line the above Becke’88 functional (113) can be further rearranged in a so called Xα-Becke88 form [88]:

(123) $$K^{XB88}=\alpha_{XB}\sum_{\sigma }\int \rho_{\sigma }^{4/3}(\text{r})\left[2^{1/3}+\frac{x_{\sigma }^{2}(\text{r})}{1+6\beta_{XB}x_{\sigma }(\text{r})\sinh^{-1}x_{\sigma }(\text{r})}\right]d\text{r}$$

where the parameters α*_XB_* and β*_XB_* are to be determined, as usually, throughout atomic fitting; it may lead with a new workable valuable density functional in exchange family.

### 4.3. Density functionals of correlation energy

The first and immediate definition of energy correlation may be given by the difference between the exact and Hartree-Fock (HF) total energy of a poly-electronic system [89]:

(124) $$E_{c}[\rho ]=E[\rho ]-E_{HF}[\rho ]$$

Instead, in density functional theory the correlation energy can be seen as the gain of the kinetic and electron repulsion energy between the full interacting (λ = 1) and non-interacting (λ = 0) states of the electronic systems [90]:

(125) $$E_{c}^{\lambda }[\rho ]=〈\psi^{\lambda }|\left(\hat{T}+\lambda {\hat{V}}_{ee}\right)|\psi^{\lambda }〉-〈\psi^{\lambda =0}|\left(\hat{T}+\lambda {\hat{V}}_{ee}\right)|\psi^{\lambda =0}〉.$$

In this context, taking the variation of the correlation energy (125) respecting the coupling parameter λ [91, 92],

(126) $$\lambda \frac{\partial E_{c}^{\lambda }[\rho ]}{\partial \lambda }=E_{c}^{\lambda }[\rho ]+\int \rho (\text{r})\text{r}\cdot \nabla \frac{\delta E_{c}^{\lambda }[\rho ]}{\delta \rho (\text{r})}d\text{r},$$

by employing it through the functional differentiation with respecting the electronic density,

(127) $$\lambda \frac{\partial V_{c}^{\lambda }[\rho ]}{\partial \lambda }-V_{c}^{\lambda }[\rho ]=\text{r}\cdot \nabla V_{c}^{\lambda }+\int \rho ({\text{r}}_{1}){\text{r}}_{1}\cdot \nabla_{1}\frac{\delta^{2}E_{c}^{\lambda }[\rho ]}{\delta \rho (\text{r})\delta \rho ({\text{r}}_{1})}d{\text{r}}_{1},$$

one obtains the equation to be solved for correlation potential $V_{c}^{\lambda }=\delta E_{c}^{\lambda }[\rho ]/\delta \rho$; then the correlation energy is yielded by back integration:

(128) $$E_{c}^{\lambda }[\rho ]=\int V_{c}^{\lambda }(\text{r},[\rho ])\rho (\text{r})d\text{r}$$

from where the full correlation energy is reached out by finally setting λ = 1.

When restricting to atomic systems, i.e. assuming spherical symmetry, and neglecting the last term of the correlation potential equation above, believed to be small [90], the equation to be solved simply becomes:

(129) $$\lambda \frac{\partial V_{c}^{\lambda }[\rho ]}{\partial \lambda }-V_{c}^{\lambda }[\rho ]=r\nabla V_{c}^{\lambda }$$

that can really be solved out with the solution:

(130) $$V_{c}^{\lambda }=A_{p}\lambda^{p+1}r^{p}$$

with the integration constants *A_p_* and *p*.

However, since the equation (129) is a homogeneous differential one, the linear combination of solutions gives a solution as well. This way, the general form of correlation potential looks like:

(131) $$V_{c}^{\lambda }=\sum_{p}A_{p}\lambda^{p+1}r^{p}.$$

This procedure can be then iterated by taking further derivative of (127) with respect to the density, solving the obtained equation until the second order correction over above first order solution (131),

(132) $$V_{c}^{\lambda }=\sum_{p1}A_{p1}\lambda^{p1+1}r^{p1}+\sum_{p2}A_{p2}\lambda^{2p2+1}r^{p2}〈r^{p2}\rho 〉.$$

By mathematical induction, when going to higher orders the *K*-truncated solution is iteratively founded as:

(133) $$V_{c}^{\lambda }=\sum_{p}\sum_{k=1}^{K}A_{pk}\lambda^{pk+1}r^{p}{〈r^{p}\rho 〉}^{k-1}$$

producing the λ-related correlation functional:

(134) $$E_{c}^{\lambda }[\rho ]=\sum_{p}\sum_{k=1}^{K}\frac{1}{k}A_{pk}\lambda^{pk+1}{〈r^{p}\rho 〉}^{k}$$

and the associate full correlation energy functional (λ=1) expression:

(135) $$E_{c}[\rho ]=\sum_{p}\sum_{k=1}^{K}\frac{1}{k}A_{pk}{〈r^{p}\rho 〉}^{k}.$$

As an observation, the correlation energy (135) supports also the immediate not spherically (molecular) generalization [90]:

(136) $$E_{c}[\rho ]=\sum_{lmn}\sum_{k=1}^{K}\frac{1}{k}A_{lmnk}{〈x^{1}x^{m}x^{n}\rho 〉}^{k}.$$

Nevertheless, for atomic systems, the simplest specialization of the relation (135) involves the simplest density moments 〈ρ〉 = *N* and 〈*r*ρ〉 that gives:


(137) $$E_{c}[\rho ]=A_{c0}N+A_{c1}〈r\rho 〉.$$

Unfortunately, universal atomic values for the correlation constants *A_c_*_0_ and *A_c_*_1_ in (137) are not possible; they have to be related with the atomic number Z that on its turn can be seen as functional of density as well. Therefore, with the settings


(138) $$A_{c0}=C_{c0}\ln Z\;,\;A_{cl}=C_{c1}Z$$

the fitting of (137) with the HF related correlation energy (124) reveals the atomic-working correlation energy with the form [90]:

(139) $$E_{c}=-0.16569N\ln Z+0.0000401Z〈r\rho 〉$$

The last formula is circumvented to the high-density total correlation density approaches rooting at their turn on the Thomas-Fermi atomic theory. Very interesting, the relation (139) may be seen as an atomic reflection of the (solid state) high density regime ( *r_s_* <1) given by Perdew et al. [75, 80, 93–96]:

(140) $$E_{c}^{PZ\infty }[\rho ]=\int d\text{r}\rho (\text{r})(-0.048-0.0116r_{s}+0.0311\ln r_{s}+0.0020r_{s}\ln r_{s})$$

in terms of the dimensionless ratio

(141) $$r_{s}=\frac{r_{0}}{a_{0}}$$

between the Wigner-Seitz radius *r*_0_ = (3/4πρ)^1/3^ , see relation (52), and the first Bohr radius *a_0_* = ħ^2^ / *me*^2^.

Instead, within the low density regime ( *r_s_* ≥ 1) the first approximation for correlation energy goes back to the Wigner jellium model of electronic fluid in solids thus providing the LDA form [97, 98]:

(142) $$E_{c}^{W-LDA}[\rho ]=\int \varepsilon_{c}[\rho (\text{r})]\rho (\text{r})d\text{r},$$

where

(143) $$\varepsilon_{c}[\rho (\text{r})]=-\frac{0.44}{7.8+r_{s}}$$

is the correlation energy per particle of the homogeneous electron gas with density ρ, see relations (55) and (56).

However, extended parameterization of the local correlation energy may be unfolded since considering the fit with an LSDA (ρ_↑_ and ρ_↓_) analytical expression by *Vosko, Wilk and Nusair* (VWN) [99],

(144) $$E_{c}^{VWN}[\rho_{↑},\rho_{↓}]=\int \varepsilon_{c}[\rho_{↑}(\text{r}),\rho_{↓}(\text{r})]\rho (\text{r})d\text{r}$$

while further density functional *gradient corrected Perdew* (GCP) expansion will look like:

(145) $$E_{c}^{GCP}[\rho_{↑},\rho_{↓}]=\int dr\varepsilon_{c}[\rho_{↑}(\text{r}),\rho_{↓}(\text{r})]\rho (\text{r})+\int d\text{r}B[\rho_{↑}(\text{r}),\rho_{↓}(\text{r})]{\left|\nabla \rho (\text{r})\right|}^{2}+\ldots$$

where the Perdew recommendation for the gradient integrant has the form [100]:

(146) $$B_{c}^{P}[\rho_{↑}(\text{r}),\rho_{↓}(\text{r})]=B_{c}[\rho ]\frac{\text{exp}\left(-b[\rho ]f\left|\nabla \rho \right|\rho^{-7/6}\right)}{d(m)}$$

with

(147) $$B_{c}[\rho ]=\rho^{-4/3}C[\rho ]$$

being the electron gas expression for the coefficient of the gradient expansion. The normalization in (146) is to the spin degeneracy:

(148) $$\begin{array}{c} d(m)=2^{1/3}{\left[{\left(\frac{1+m}{2}\right)}^{5/3}+{\left(\frac{1-m}{2}\right)}^{5/3}\right]}^{1/2},\\ m=\frac{\rho_{↑}-\rho_{↓}}{\rho },\;\rho =\rho_{↑}+\rho_{↓}, \end{array}$$

while the exponent containing functional

(149) $$b[\rho ]={\left(9\pi \right)}^{1/6}\frac{C[\rho \to \infty ]}{c[\rho ]}$$

is written as the ratio of the asymptotic long-range density behavior to the current one, and is controlled by the cut-off *f* exponential parameter taking various values depending of the fitting procedures it subscribes (0.17 for closed shells atoms and 0.11 for Ne particular system [101, 102]).

More specifically, we list bellow some nonlocal correlation density functionals in the low density (gradient corrections over LDA) regime:

- the Rasolt and Geldar paramagnetic case (ρ_↑_ = ρ_↓_ = ρ/*2*) is covered by correlation energy [101, 103]:
(150) $$E_{c}^{RG}[\rho ]=c_{1}+\frac{c_{2}+c_{3}r_{s}+c_{4}r_{s}^{2}}{1+c_{5}r_{s}+c_{6}r_{s}^{2}+c_{7}r_{s}^{3}}$$

with *c*_1_=1.667·10^−3^, *c*_1_=2.568·10^−3^, *c*_3_=2.3266·10^−2^, *c*_4_=7.389·10^−6^, *c*_5_=8.723, *c*_6_=0.472, *c*_7_=7.389·10^−2^ (in atomic units).

- The gradient corrected correlation functional reads as [102, 104]:
(151) $$\begin{array}{c} E_{c}^{GC}=\int d\text{r}\varepsilon_{c}[\rho_{↑},\rho_{↓}]\rho (\text{r})+\int d\text{r}B_{c}^{p}{\left[\rho_{↑},\rho_{↓}\right]}_{c[\rho ]=\sqrt{2\pi }/4{\left({6\pi }^{2}\right)}^{4/3},f=0.17}|\nabla \rho (\text{r}){|}^{2}+\\ +9\frac{\pi }{4{\left(6\pi^{2}\right)}^{4/3}}{\left(0.17\right)}^{2}\int d\text{r}\left(|\nabla \rho_{↑}{|}^{2}\rho_{↑}^{-4/3}+|\nabla \rho_{↓}{|}^{2}\rho_{↓}^{-4/3}\right) \end{array}$$
- The *Lee, Yang, and Parr* (LYP) functional within Colle-Salvetti approximation unfolds like [105]:
(152) $$\begin{array}{c} E_{c}^{LYP}=-a_{c}b_{c}\int d\text{r}\gamma (\text{r})\xi (\text{r})\left(\underset{\sigma }{\Sigma }\rho_{\sigma }(\text{r})\underset{i}{\Sigma }|\nabla \varphi_{i\sigma }(\text{r}){|}^{2}-\frac{1}{4}\underset{\sigma }{\Sigma }\rho_{\sigma }(\text{r})\text{Δ}\rho_{\sigma }(\text{r})-\frac{1}{4}|\nabla \rho (\text{r}){|}^{2}+\frac{1}{4}\rho (\text{r})\text{Δ}\rho (\text{r})\right)\\ -a_{c}\int d\text{r}\frac{\gamma (\text{r})}{\eta (\text{r})}\rho (\text{r}) \end{array}$$

where

(153) $$\gamma (\text{r})=4\frac{\rho_{↑}(\text{r})\rho_{↓}(\text{r})}{\rho {\left(\text{r}\right)}^{2}},\;\eta (\text{r})=1+d_{c}\rho {\left(\text{r}\right)}^{-1/3},\;\xi (\text{r})=\frac{\rho {\left(\text{r}\right)}^{-5/3}}{\eta (\text{r})}\exp[-c_{c}\rho {\left(\text{r}\right)}^{-1/3}]$$

and the constants: *a_c_*=0.04918, *b_c_*=0.132, *c_c_*=0.2533, *d_c_*=0.349.

- The open-shell (OS) case provides the functional [98]:
(154) $$E_{c}^{OS}=\int d\text{r}\frac{a_{s}\rho (\text{r})+b_{s}|\nabla \rho (\text{r})|\rho {\left(\text{r}\right)}^{-1/3}}{c_{s}+d_{s}(|\nabla \rho_{↑}|\rho_{↑}^{-4/3}+|\nabla \rho_{↓}|\rho_{↓}^{-4/3})+r_{s}}\sqrt{1-\zeta^{2}}$$

with the spin-dependency regulated by the factor ζ = (ρ_↑_ − ρ_↓_)/(ρ_↑_ + ρ_↓_) approaching zero for closed-shell case, while the specific coefficients are determined through a scaled-minimization procedure yielding the values: *a_s_*=−0.74860, *b_s_*=−0.06001, *c_s_*=3.60073, *d_s_*=0.900000.

- Finally, Perdew and Zunger (PZ) recommend the working functional [106]:
(155) $$E_{c}^{PZ0}[\rho ]=\int d\text{r}\rho (\text{r})\frac{\alpha_{p}}{1+\beta_{1p}\sqrt{r_{s}}+\beta_{2p}r_{s}}$$

with the numerical values for the fitting parameters founded as: α*_p_*=−0.1423, β*_1p_*=1.0529, β*_2p_*=0.3334.

### 4.4. Density functionals of exchange-correlation energy

Another approach in questing exchange and correlation density functionals consists in finding them both at once in what was defined as exchange-correlation density functional (29). In this regard, following the Lee and Parr approach [107], the simplest starting point is to rewrite the inter-electronic interaction potential

(156) $$V_{ee}\iint \frac{\rho_{2}({\text{r}}_{1},{\text{r}}_{2})}{r_{12}}d{\text{r}}_{1}d{\text{r}}_{2}$$

and the classical (Coulombic) repulsion

(157) $$J=\frac{1}{2}\iint \frac{\rho ({\text{r}}_{1})\rho ({\text{r}}_{2})}{r_{12}}d{\text{r}}_{1}d{\text{r}}_{2},$$

appeared in the formal exchange energy (*V_ee_* – *J*) in (21), by performing the previously introduced coordinate transformation (79), followed by integration of the averaged pair and coupled densities (denoted with over-bars) over the angular components of **s**:

(158) $$V_{ee}=4\pi \int dr\int sds{\bar{\rho }}_{2}(\text{r},s),$$

(159) $$J=2\pi \int d\text{r}\int sds\bar{\rho (\text{r}+\text{s}/2)\rho (\text{r}-\text{s}/2)}.$$

Now, the second order density matrix in (158) can be expressed as

(160) $${\bar{\rho }}_{2}(\text{r},s)=\frac{1}{2}\bar{\rho (\text{r}+\text{s}/2)\rho (\text{r}-\text{s}/2)}[1+F_{1}(\text{r},s)]$$

with the help of the introduced function *F*_1_ (**r**, *s*) carrying the form

(161) $$F_{1}(\text{r},s)=-\frac{\exp[-\alpha (\text{r})s]}{1+\alpha (\text{r})}\{1+{\left[\alpha (\text{r})s\right]}^{2}F_{2}(\text{r},s)\}$$

so that the cusp condition for ρ̄_2_ (**r**, *s*)

(162) $${\frac{\partial \ln{\bar{\rho }}_{2}(\text{r},s)}{\partial s}|}_{s=0}=1$$

to be satisfied for a well behaved function of a Taylor series expansion type

(163) $$F_{2}(\text{r},s)=\sum_{k=0}^{\infty }a_{k}(\text{r}){\left[\alpha (\text{r})s\right]}^{k}$$

when α(**r**) stands for a suitable function of **r** as well, see bellow.

On the other side, the average $\bar{\rho (\text{r}+\text{s}/2)\rho (\text{r}-\text{s}/2)}$ in (159) and (160) supports a Taylor expansion [108]:

(164) $$\bar{\rho (\text{r}+\text{s}/2)\rho (\text{r}-\text{s}/2)}=\rho^{2}(\text{r})\left[1-\frac{2\tau_{w}(\text{r})}{3\rho (\text{r})}s^{2}+\ldots \right]$$

with

(165) $$\tau_{w}(\text{r})=\frac{1}{8}\frac{|\nabla \rho (\text{r}){|}^{2}}{\rho (\text{r})}-\frac{1}{8}\nabla^{2}\rho (\text{r})$$

being the Parr modified kinetic energy of Weizsäcker type [9].

Inserting relations (158)-(165) in (*V_ee_* – *J*) difference it is eventually converted from the “genuine” exchange meaning into practical exchange-correlation energy characterized by the density functional form:

(166) $$\begin{array}{c} E_{xc}=2\pi \int d\text{r}\int sds\;\bar{\rho (\text{r}+\text{s}/2)\rho (\text{r}-\text{s}/2)}F_{1}(\text{r},s)\\ =-2\pi \int d\text{r}\frac{\rho^{2}(\text{r})}{1+\alpha (\text{r})}\int ds\;s\exp[-\alpha (\text{r})s]\left\{1-\frac{2\tau_{w}(\text{r})}{3\rho (\text{r})}s^{2}+...\right\}\left\{1+{\left[\alpha (\text{r})s\right]}^{2}\overset{\infty }{\underset{k=0}{\Sigma }}a_{k}(\text{r})s{]}^{k}\right\} \end{array}$$

Making use of the two possible multiplication of the series in (166), i.e. either by retaining the α(**r**) containing function only or by including also the density gradient terms in the first curled brackets, thus retaining also the term containing τ*_w_* (**r**) function, the so called *I-xc or II-xc type functionals* are respectively obtained.

Now, laying aside other variants and choosing the simple (however meaningfully) density dependency

(167) $$\alpha (\text{r})=k\rho^{1/3}(\text{r}),\;k=\text{constant}$$

the provided exchange-correlation functionals are generally shaped as [107]:

(168) $$\begin{array}{c} E_{sc}^{I}=-\frac{1}{k^{2}}\int d\text{r}\rho^{4/3}(\text{r})\frac{A_{sc}(\text{r})}{1+k\rho^{1/3}(\text{r})},\\ E_{xc}^{II}=-\frac{1}{k^{2}}\int d\text{r}\frac{\rho^{4/3}(\text{r})}{1+k\rho^{1/3}(\text{r})}\left[B_{xc}(\text{r})+\frac{2}{3}\frac{\tau_{w}(\text{r})}{\rho^{5/3}(\text{r})}C_{xc}(\text{r})\right]. \end{array}$$

These functionals are formally exact for any κ albeit the resumed functions *A_xc_*(**r**), *B_xc_*(**r**), and *C_xc_*(**r**) are determined for each particular specialization.

Going now to the specific models, let’s explore the type I of exchange-correlation functionals (168). Firstly, they can further undergo simplification since the reasonable (atomic) assumption according which

(169) $$k\rho^{1/3}(\text{r})\ll 1,\forall \text{r}.$$

Within this frame the best provided model is of *X*α-Padé approximation type, containing *N*-dependency [107]:

(170) $$E_{xc}^{I(X\alpha )}=-a_{0}^{X\alpha }\frac{1+a_{1}^{X\alpha }/N}{1+a_{2}^{X\alpha }/N}\int \rho^{4/3}(\text{r})d\text{r}$$

with *a*_0_*^Xα^*=0.7475, *a*_1_*^Xα^*=17.1903, and *a*_2_*^Xα^*=14.1936 (atomic units).

When the condition (169) for κ is abolished the Wigner-like model results, again, having the best approximant exchange-correlation model as the Padé form [107]:

(171) $$E_{xc}^{I(Wig)}=-a_{0}^{Wig}\frac{1+a_{1}^{Wig}/N}{1+a_{2}^{Wig}/N}\int \frac{\rho^{4/3}(\text{r})}{1+k^{I(Wig)}\rho^{1/3}(\text{r})}d\text{r}$$

with *a*_0_*^Wig^*=0.76799, *a*_1_*^Wig^*=17.5943, *a*_2_*^Wig^*=14.8893, and κ*^I (Wig)^*=4.115·10^−3^ (atomic units).

Turning to the II-type of exchange-correlation functionals, the small density condition (169) delivers the gradient corrected *X*α model, taking its best fitting form as the *N*-dependent Padé approximant [107]:

(172) $$E_{xc}^{II(X\alpha )}=-b_{0}^{X\alpha }\frac{1+b_{1}^{X\alpha }/N}{1+b_{2}^{X\alpha }/N}\int \rho^{4/3}(\text{r})d\text{r}-c_{0}^{X\alpha }\int \rho^{-1/3}(\text{r})\tau_{w}(\text{r})d\text{r}$$

with *b*_0_*^Xα^*=0.7615, *b*_1_*^Xα^*=1.6034, *b*_2_*^Xα^*=2.1437, and *c*_2_*^Xα^*=6.151·10^−2^ (atomic units), while when laying outside the (169) condition the gradient corrected Wigner-like best model is proved to be without involving the *N*-dependency [107]:

(173) $$E_{sc}^{II(Wig)}=-b_{0}^{Wig}\int \frac{\rho^{4/3}(\text{r})}{1+k^{II(Wig)}\rho^{1/3}(\text{r})}d\text{r}-c_{0}^{Wig}\int \frac{\rho^{-1/3}(\text{r})\tau_{w}(\text{r})}{1+k^{II(Wig)}\rho^{1/3}(\text{r})}d\text{r}$$

with *b*_0_*^Wig^*=0.80569, *c*_0_*^Wig^*=3.0124·10^−3^, and κ*^II(Wig)^*=4.0743·10^−3^ (atomic units).

Still, a Padé approximant for the gradient-corrected Wigner-type exchange-correlation functional exists and it was firstly formulated by Rasolt and Geldar [103] with the working form [109]:

(174) $$E_{xc}^{RG}=E_{xc}^{LDA(orX\alpha )}+\int B_{xc}^{RG}[\rho (\text{r})]\frac{|\nabla \rho (\text{r}){|}^{2}}{\rho^{1/3}(\text{r})}d\text{r}$$

with *B_xc_^RG^* given with the Padé form:

(175) $$B_{xc}^{RG}[\rho (\text{r})]=-1\times 10^{-3}c_{1}^{RG}\frac{1+c_{2}^{RG}r_{s}+c_{3}^{RG}r_{s}^{2}}{1+c_{4}^{RG}r_{s}+c_{5}^{RG}r_{s}^{2}+c_{6}^{RG}r_{s}^{3}}$$

having the fitted coefficients *c*_1_*^RG^*=2.568, *c*_2_*^RG^*=9.0599, *c*_3_*^RG^*=2.877·10^−3^, *c*_4_*^RG^*=8.723, *c*_5_*^RG^*=0.472, and *c_3_^RG^*=7.389·10^−2^ (atomic units). Some studies also consider the nonlocal correction in (174) premultiplied by the 10/7 factor which was found as appropriate procedure for atomic systems.

Finally, worth noting the Tozer and Handy general form for exchange-correlation functionals viewed as a sum of products of powers of density and gradients [110]:

(176) $$E_{xc}^{TH}=\int F_{xc}(\rho_{↑},\rho_{↓},\zeta_{↑↓})d\text{r}$$

with

(177) $$F_{xc}=\sum_{abcd}\omega_{abcd}R^{a}S^{b}X^{c}Y^{d}=\sum_{abcd}\omega_{abcd}f_{abcd}(\text{r})$$

where

(178) $$\begin{array}{c} R^{a}=\rho_{↑}^{a}+\rho_{↓}^{a},\\ S^{b}=m^{2b}\ldots \;\text{see}\;\text{equation}\;\text{(148)}\;\text{for }m\;\;definition,\\ X^{c}=\frac{\zeta_{↑}^{c}+\zeta_{↓}^{c}}{2\rho^{4c/3}},\\ Y^{d}={\left(\frac{\zeta_{↑}^{2}+\zeta_{↓}^{2}-2\zeta_{↑↓}}{\rho^{8/3}}\right)}^{d} \end{array}$$

and

(179) $$\zeta_{↑}=\left|\nabla \rho_{↑}\right|,\;\zeta_{↓}=\left|\nabla \rho_{↓}\right|,\;\zeta_{↑↓}=\nabla \rho_{↑}\cdot \nabla \rho_{↓},\;\rho =\rho_{↑}+\rho_{↓}.$$

The coefficients ω*_abcd_* of (177) are determined through minimization procedure involving the associated exchange-correlation potentials $V_{xc↑(↓)}^{abcd}(\text{r})=\delta f_{abcd}(\text{r})/\delta \rho_{↑(↓)}(\text{r})$ in above (176) functional. The results would depend upon the training set of atoms and molecules but presents the advantage of incorporating the potential information in a non-vanishing asymptotical manner, with a semi-empirical value. Moreover, its exact asymptotic exchange-correlation potential equals chemical hardness [9, 12, 14, 20] for open-shell being less than that for closed shell systems, thus having the merit of including chemical hardness as an intrinsic aspect of energetic approach, a somewhat absent aspect from conventional functionals so far.

However, since electronegativity and chemical hardness closely relate with chemical bonding, their relation with the total energy and component functionals is in next at both conceptual and applied levels explored.

## 5. Testing (χ, η) Quadratic Dependency Among Several Energetic Density Functionals

### 5.1. Proof of the E=E(χ,η) quadratic dependency

Employing the Kohn-Sham equation (33) for the (reactivity) equalized chemical potential (minus electronegativity) eigenvalues, among atomic or molecular one-electronic orbitals

(180) $$\mu_{i}=\mu_{j}=\ldots =\mu ,$$

in quantum mechanically sense, we immediately get:

(181) $$\mu =\frac{\int \varphi^{•}(\text{r})\left[-\frac{1}{2}\nabla^{2}+V_{eff}\right]\varphi (\text{r})d\text{r}}{\int \varphi^{•}(\text{r})\varphi (\text{r})d\text{r}}=\frac{\int \varphi^{•}(\text{r})\hat{H}\varphi (\text{r})d\text{r}}{\int \varphi^{•}(\text{r})\varphi (\text{r})d\text{r}}=\frac{E}{N}$$

For proper characterization of the chemical systems the quantum (Ehrenfest) version of the fundamental Newtonian law [111], linking the observed force with the minus of the potential gradient *F* = −∇*V*, is here written for chemical reactions modeled throughout the charge transfer along the reaction path as:

(182) $$F_{\mu }=-\frac{d}{dN}(\mu ).$$

Now, combining the last two equations projected on the reaction path we successively obtain:

(183) $$\begin{array}{ll} F_{\mu } & =-\frac{d}{dN}\left(\frac{E}{N}\right)\\ & =\frac{E}{N^{2}}-\frac{1}{N}{\left(\frac{dE}{dN}\right)}_{V}. \end{array}$$

At this point, taking for the derivative in (183) the finite correspondence in what regarding the chemical potential formal (absolute) definition (181),

(184) $${\left(\frac{dE}{dN}\right)}_{V}=\mu =\frac{E}{N},$$

the chemical potential energy equation is unfolded as:

(185) $$E_{\mu }=\mu N+F_{\mu }N^{2}.$$

The remaining issue is to clarify the chemical potential related force meaning in above equation. In this respect, by considering the electronegativity-chemical potential relationship (19), the associate electronegativity energy equation becomes:

(186) $$E_{\chi }=-\chi N+F_{\chi }N^{2}$$

while the involved electronegativity related force is seen as the reactive force of the chemical potential (182),

(187) $$F_{\chi }=-F_{\mu }$$

recovering the chemical hardness index:

(188) $$F_{\chi }=-\frac{d}{dN}(\chi )=\eta .$$

With these considerations, the chemical reactivity energy equation (185) is finally displayed as

(189) $$E=-\chi N+\eta N^{2}$$

equally supporting the differential (statistical, thermodynamical) variant:

(190) $$dE=-\chi dN+\eta {\left(dN\right)}^{2}$$

based on the above finite-differential (184) equivalence between the total (*E*, *N*) or exchanged (*dE*, *dN*) energy and charge, respectively.

What there was actually proofed is that the quadratic chemical reactivity equations for total energy in both finite and differential fashions may be derived employing the Kohn-Sham (as Schrödinger reminiscence) equation for chemical potential eigenvalue combined with the chemical quantum version of the Ehrenfest theorem involving the force concept and its active-reactive peculiar property for the chemical potential and electronegativity, respectively. No particular assumptions were considered being all above arguments only on first principles grounded. Thus, the exposed demonstration is of general value cutting much discussion in the last decades on the viability of the second order truncation in the total energy expansion in terms of chemical reactivity indices, viz. electronegativity and chemical hardness concepts.

However, a computational test for this behavior is in next addressed.

### 5.2. Atomic and molecular analysis of the energetic quadratic bilinear (χ, η) dependency

The general bivariate equation linking the energy (various) functionals with electronegativity and chemical hardness, either for atomic and molecular systems, works out with form:

(191) E=a+bχ+cη

with coefficients *a*, *b*, and *c* being determined throughout consecrated statistical analysis methods [112]. The result of such correlation will lead with two kind of information:

- the degree of correlation itself between the employed energy functional and the couple of electronegativity-chemical hardness structural indices; this is measured by *the standard correlation factor* [113]:
(192) $$r=\sqrt{1-\frac{\underset{i}{\Sigma }{\left(y_{iINPUT}-y_{iFIT}\right)}^{2}}{\underset{i}{\Sigma }{\left(y_{iINPUT}-\bar{y_{iINPUT}}\right)}^{2}}}$$
- the degree of parabolic dependency by checking whether the chemical hardness coefficient (*c*) is the square of the electronegativity coefficient (*b*) thus giving the opportunity of introducing the so-called *sigma-pi reactivity index*
(193) $$\sigma_{\pi }=sign(b)\frac{c}{b^{2}}\overset{\text{parabolic}\;E=E(\chi ,\eta )}{\to }-1$$

At this point worth nothing that as σ_π_ → −1 as better the energy fulfils the correlation shape with the established parabolic equation (189). The *b* and *c* signs open further discussion among the allowed combinations for the correlation equation (191). Firstly, let’s observe that the signs of *b* and *c* give information about the signs of electronegativity and chemical hardness, respectively. Then, while considering the finite-difference approximations to the electronegativity and chemical hardness definitions, in a Koopmans theorem environment [114], they take the working forms [9, 14, 115, 116]:

(194) $$\chi \equiv -{\left(\frac{\partial E_{N}}{\partial N}\right)}_{V(r)}≅-\frac{E_{N+1}-E_{N-1}}{2}=\frac{\left(E_{N-1}-E_{N})+(E_{N}-E_{N+1}\right)}{2}=\frac{IP+EA}{2}≅-\frac{HOMO+LUMO}{2}$$

(195) $$\eta ={\left(\frac{\partial^{2}E_{N}}{\partial N^{2}}\right)}_{V(r)}≅E_{N+1}-2E_{N}+E_{N-1}=IP-EA≅LUMO-HOMO$$

in terms of lowest unoccupied molecular orbital (*LUMO*) and highest occupied molecular orbital (*HOMO*) energies, although similar relations (in terms of ionization potential *IP* and electron affinity *EA*) hold for atomic systems as well. Nevertheless, the expressions (194) and (195) help in deciding that only the (−, +) and (+, +) combinations for coefficients (*b*, *c*) are allowed, based on the fact that only maximum hardness values (i.e. positive values of *c* coefficient) are in accordance with the reactivity criteria of maximum hardness principle [117–119]; in other words, the correlation combination carrying negative values for chemical hardness coefficient in (191) are less physically probably since that would imply that *EA*>0 and *LUMO*<0, for atoms and molecules, respectively.

Finally, the sigma-pi index (193) can be used in defining another reactivity index, namely *the efficiency index*:

(196) $$\sum_{\text{Π}}=|\sigma_{\pi }|\overset{parabolic\;E=E(\chi ,\eta )}{\to }1$$

measuring the power of electronic exchange characterizing the chemical reactivity which is maximized by the exact parabolic dependence of the energetic functional respecting the electronegativity-chemical hardness jointly influence.

With these, the Tables I–III and IV–V display the tested energetic functionals against various electronegativity and chemical hardness scales for selected atoms and molecules, while the Tables VI and VII show their quantitative (χ & η) structural– (energy functionals) property relationships (QSPR), respectively.

At a glance, Tables VI and VII reveals that the parabolic energetic dependency (189) on the electronegativity and chemical hardness indices is not exactly recovered since all sign combinations in the coefficients *c* & *b* as well as poor correlation itself with the (χ, η) indices is revealed, i.e. widely deviating from the optimum (−,+) and maximum correlation coefficient of (192).

However, for atomic analysis, Table VI gives us that the finite-difference scale of electronegativity and chemical hardness provides invariable right (−, +) sign combination in coefficients (*b*, *c*), excepting kinetic energy functionals cases, however revealing both scarce reactive efficiency (196) and bilinear correlation (192) in almost all treated cases. Notably, the higher reactivity efficiency on studied atomic system was furnished by the correlation energy merely than the total one, yet with a low correlation factor.

The situation is somehow changed for molecular systems, see Table VII, where, albeit not better records are noted in the sense of the maximum reactive efficiency (196), again excepting the correlation functional case rather than the total energy, a higher correlation factor in semiclassical treatment of the electronegativity and chemical hardness scales is obtained; such behavior was recently validated for hard and soft acids and bases reactions as well [24]. At the same time, the kinetic energy functionals do not fit at all with standard (−,+) correlation scheme for signs of (*c*, *b*) coefficients in (191).

**Figure 5.**
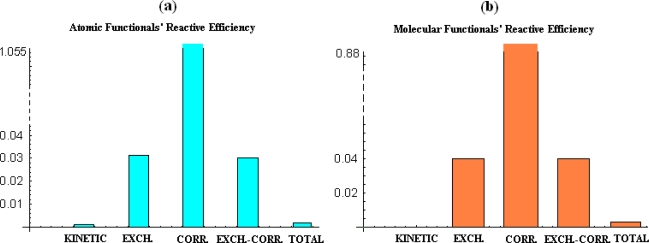
Charts of energetic functionals showing their reactive efficiency (196) as the height of bars for the parabolic expansion and of their statistical correlation (192) as the width of bars, in terms of electronegativity and chemical hardness, resuming atomic analysis of Tables I–III - in (a), and molecular one from Tables IV and V - in (b), by employing representative values of Tables VI and VII (highlighted on grey background), respectively.

Finally, in Figure 5 the atomic and molecular parabolic reactivity efficiencies (196) of various energetic functionals are plotted as charts with the widths emphasizing the bilinear electronegativity & chemical hardness correlation values of (192) factor as abstracted from Tables VI and VII. The comparative behavior reveals many interesting features to be taking into account for further conceptual and computational studies on quantum chemistry:

- the correlation energy appears to provide acceptable parabolic shapes in both atomic and molecular cases, with better bilinear regression for molecular analysis, while strongly depending on the electronegativity and chemical hardness atomic models and scales;
- the kinetic energy, while displaying poor parabolic shape at atomic level behaves with negative chemical hardness in molecular systems, probably due the positive contribution in bonding that compete with stabilization (localization) of the electrons within internuclear basin;
- exchange and exchange-correlation functionals reveal similar reactive (parabolic) efficiency as well as close bivariate regression correlation factors for both atomic and molecular cases, leaving with the impression that the exchange contribution is dominant in exchange-correlation functionals since cancelling somehow the behavior of the correlation part of the functional.
- overall, the total energy, although with correlation factors in the range of its components’ regressions does not fit with parabolic reactive theoretical prescription (189), at least for present employed set of atoms and molecules.

However, all revealed parabolic features have appeared on the statistical correlation basis and not upon an individual atomic or molecular analysis. At this point we can refer to studies [126] that clearly illustrate the individual parabolic behavior of the total energy against the total number of electrons. On the other side, the systematic focus on atomic and molecular large class of systems should provide more or less the same phenomenological results.

The present dichotomy, while confronting the conceptual and computational behavior of the energetic functionals respecting the parabolic bilinear expansion in terms of electronegativity and chemical hardness, gives the indication that the derivation of the energetic functionals (and of the total energy in special) starting from electronegativity and chemical hardness functionals [14, 123], may eventually conclude with better energy correlation both as parabolic shape and statistical regression with fundamental (χ, η) couple of reactivity indices. Otherwise, we will be forced to admit that the density functionals do not properly model the reactivity through a parabolic fit in total or exchanged electrons of the involved systems at least in a statistical sense.

## 6. Conclusions

Why another quantum theory of the matter? Why the *Density Functional Theory*? Its pioneers and promoters, Walter Kohn, Axel Becke and Robert G. Parr – just to name a few, affirm that modeling electronic density features in 3D real space produces much benefits in both conceptually understanding of bonding as well in electronic localization with a bonding impact for the concerned *N*-many-electronic systems [127–129].

The present paper aimed to unitarily presents the quantum fundaments of DFT as well to prospect directions in electronic bonding and reactivity characterization by using the density functionals and localization functions. Such theoretical tools allow the rationalization of the electronic structures while offering an analytical understanding and prediction of the experimental data along of the modeled reality.

In DFT, the density and their combinations in the density functionals of the total energy plays a primordial role. It fulfills the *N*-contingency, assures the total energy minimization, influences the different levels of approximation, i.e. local density or gradient density frameworks, controls the bonding through electronic localization functions, and decides upon reactivity through the electronic exchange relating the electronegativity and chemical hardness indices. Studying the electronic density properties of electronic systems guaranties the universal treatment of whatever systems, no matter how rich in electrons is, from atoms, to molecules, to solids and aggregates.

Going to link the inner structural information with the manifested chemical reactivity the various energetic density functionals of atoms and molecules were presented, emphasizing on different levels of approximation and of quantum containing information. Nevertheless, their values were assumed merely as properties of particular systems when combined with associate reactive peculiarities quantified by electronegativity and chemical hardness. A theoretical link, based on first quantum mechanical principles, was also provided at a level of chemical potential-chemical force couple for the electronegativity and chemical hardness actions, respectively.

Actually, the parabolic shape of the energetic dependence on electronegativity and chemical hardness ( *E* = *a* + *b*χ + *c*η) is theoretically proofed with the help of potential-force physical picture of interaction and dynamics, while the computational cases were treated throughout the introduced sigma-pi index σ_π_ = *sign*(*b*)*c* / *b*^2^ or parabolic reactive efficiency ∑_Π_ = |σ_π_|.

The numerical results of such correlation on selected, however limited, set of atomic and molecular systems, were intrigued since there was found out that only the correlation functionals provide appropriate parabolic and statistical behavior among considered atoms and molecules, while all other total energy components and the total energy itself posse both surprisingly low parabolic and correlation factors with the electronegativity and chemical hardness structural parameters. Nevertheless, at atomic level the finite-difference approximation for electronegativity and chemical hardness scales was found as most suitable in providing a correct (−, +) sign combination in parabolic (*b*, *c*) coefficients, whereas molecular systems comes out within such agreement when semiclassical scale was adopted. However, the conceptual-computational parabolic reactive dichotomy of energy functionals against the reactivity indices may be eventually in future avoided by generating energy functionals directly from electronegativity and chemical hardness functionals [130]. Such paradox is to be abolished when comprehensive quantum chemical knowledge of structure and reactivity will be unfolded - step by step, from close to closer.

## Figures and Tables

**Table I.** Atomic kinetic, exchange, and correlation, energies (in hartrees) from various schemes of computations. The exact values are computed with Hartree-Fock densities.

Atoms	*Kinetic energy*				*Exchange energy*			*Correlation energy*		
	*T* *_exact_* ♣	*T*_0_ ♣	*T*_0_+*T*_2_♣	*T* *_Padé_* ♣	*K* *_exact_* ♦	*K*_0_ ♥	*K* ^B88^ ♥	*E* *_c_* *^exact^* ♦	*E* *_c_* ^(139)^ ♠	*E* *_c_* •
**He**	2.86168	2.56054	2.87850	2.87639	−1.0260	−0.884	−1.025	−0.0425	−0.0215	−0.0681
**Li**	7.43273	6.70062	7.50504	7.44941	−1.7812	−1.538	−1.775	−0.0454	−0.0486	−0.0815
**Be**	14.5730	13.1286	14.6466	14.4223	−2.6669	−2.312	−2.658	−0.0945	−0.0820	−0.1192
**B**	24.5291	22.0720	24.5228	24.2089	−3.7438	−3.272	−3.728	−0.1247	−0.1197	−0.1625
**C**	37.6886	34.0144	37.5988	37.2533	−5.0444	−4.459	−5.032	−0.1566	−0.1609	−0.2091
**N**	54.4009	49.4771	54.3852	54.0643	−6.5971	−5.893	−6.589	−0.1850	−0.2050	−0.2567
**O**	74.8094	67.8965	74.3573	74.1625	−8.1752	−7.342	−8.169	−0.2579	−0.2512	−0.3035
**F**	99.4093	90.4598	98.6429	98.6959	−10.003	−9.052	−10.02	−0.332	−0.2996	−0.3510
**Ne**	128.547	117.761	127.829	128.221	−12.108	−11.03	−12.14	−0.390	−0.3498	−0.3987
**Na**	161.859	148.809	161.093	161.718	−14.017	−12.79	−14.03	−0.398	−0.3892	−0.4137
**Mg**	199.614	184.017	198.749	199.578	−15.994	−14.61	−16.00	−0.443	−0.4351	−0.4491
**Al**	241.877	223.443	240.868	242.008	−18.069	−16.53	−18.06	−0.480	−0.4809	−0.4863
**Si**	288.854	267.315	287.659	289.139	−20.280	−18.59	−20.27	−0.521	−0.5308	−0.5308
**Cl**	459.482	426.865	457.321	460.117	−27.512	−25.35	−27.49	−0.714	−0.6901	−0.6710
**Ar**	526.817	490.017	524.289	527.617	−30.185	−27.86	−30.15	−0.787	−0.7459	−0.7190

♣ : from Ref. [71] and references therein;

♦ : from Ref. [107] and references therein;

♥ : from Ref. [84];

♠ : from eq. (139) and Ref. [90];

• : from fitting equation *E_c_*=−0.04682*N* + 0.005753<*ρ*^2/3^>−0.00096<*ρ*^1/3^>, see Ref. [90] and references therein

**Table II.** Atomic exchange-correlation and total energies (in hartrees) from various schemes of computations. The exact values are computed with Hartree-Fock densities.

Atoms	*Exchange-Correlation energy*					*Total energy*				
	*E* *_xc_* *^exact^* ♣	*E* *_xc_* *^I(Xα)^* ♦	*E* *_xc_* *^II(Xα)^* ♦	*E* *_xc_* *^I(Wig)^* ♦	*E* *_xc_* *^II(Wig)^* ♦	*E* *_tot_* *^exact^* ♥	*E* *_tot_* *^xc(RG)^* ♥	*E* *_tot_* *^xc(LDA)^* ♥	*E* *_tot_* *^BLYP^* ♠	*E* *_tot_* *^PW91^* ♠
**He**	−1.0685	−1.0604	−1.0566	−1.0633	−1.0654	−2.9042	−3.0317	−2.8601	−2.9071	−2.9000
**Li**	−1.8266	−1.8048	−1.8134	−1.8093	−1.8108	−7.4781	−7.6473	−7.3704	−7.4827	−7.4742
**Be**	−2.7614	−2.7260	−2.7522	−2.7325	−2.7342	−14.6675	−14.8911	−14.4966	−14.6615	−14.6479
**B**	−3.8685	−3.8126	−3.8415	−3.8215	−3.8177	−24.6538	−24.9158	−24.4097	−24.6458	−24.6299
**C**	−5.2010	−5.1127	−5.1338	−5.1248	−5.1121	−37.8163	−38.1305	−37.5095	−37.8430	−37.8265
**N**	−6.7821	−6.6400	−6.6440	−6.6558	−6.6321	−54.4812	−54.8681	−54.1287	−54.5932	−54.5787
**O**	−8.4331	−8.3599	−8.3405	−8.3796	−8.3450	−75.0271	−75.4597	−74.5979	−75.0786	−75.0543
**F**	−10.325	−10.327	−10.277	−10.350	−10.305	−99.741	−100.235	−99.247	−99.7581	−99.7316
**Ne**	−12.498	−12.551	−12.466	−12.579	−12.524	−128.937	−129.522	−128.403	−128.9730	−128.9466
**Na**	−14.415	−14.462	−14.382	−14.488	−14.445	−162.257	−162.862	−161.624	−162.293	−162.265
**Mg**	−16.437	−16.482	−16.424	−16.504	−16.484	−200.058	−200.705	−199.340	−200.093	−200.060
**Al**	−18.549	−18.566	−18.542	−18.583	−18.593	−242.357	−243.028	−241.533	−242.380	−242.350
**Si**	−20.801	−20.774	−20.791	−20.784	−20.830	−289.356	−290.063	−288.435	−289.388	−289.363
**Cl**	−28.226	−28.115	−28.272	−28.092	−28.281	−460.196	−461.005	−458.963	−460.165	−460.147
**Ar**	−30.972	−30.827	−31.037	−30.789	−31.035	−527.605	−528.452	−526.267	−527.551	−527.539

♣ : from Ref. [107];

♦ : from Eqs. (170)–(173) and Ref. [107];

♥ : from Ref. [109];

♠ : from Ref. [120]

**Table III.** Values (in hartrees) of the structural indices electronegativity (χ), chemical hardness (η), in finite-difference [121, 122], density functional [123], and semiclassical [124] modes of computations for atoms of Tables I and II.

Level	*Finite-Difference*		*Functional*		*Semiclassical*	
Atoms	*χ* *_FD_*	*η* *_FD_*	*χ* *_DFT_*	*η* *_DFT_*	*χ* *_SC_*	*η* *_SC_*
**He**	0.45094	0.45866	1.21132	1.66189	0.57038	0.2172
**Li**	0.11099	0.16134	0.15105	0.08784	0.00412	0.00334
**Be**	0.12606	0.21794	0.44248	0.44579	0.00893	0.0047
**B**	0.15656	0.14921	1.15362	1.34105	0.01526	0.00588
**C**	0.22933	0.18339	2.76332	2.9695	0.02279	0.00684
**N**	0.25616	0.27894	5.79566	4.91363	0.03139	0.0076
**O**	0.27894	0.22566	10.6505	5.91694	0.04072	0.00816
**F**	0.38221	0.25983	16.9129	4.37707	0.05061	0.00849
**Ne**	0.39361	0.40132	23.7119	−0.08747	0.06079	0.00864
**Na**	0.10290	0.10621	0.23153	0.18743	0.00011	0.00005
**Mg**	0.09555	0.18339	0.49871	0.53142	0.00018	0.00007
**Al**	0.11834	0.10327	1.04631	1.19882	0.00026	0.00008
**Si**	0.17200	0.12606	2.10805	2.30724	0.00036	0.00009
**Cl**	0.30577	0.17120	11.5766	7.7692	0.00074	0.00012
**Ar**	0.28299	0.29806	17.8831	9.08857	0.00088	0.00013

**Table IV.** Molecular kinetic, exchange, correlation, exchange-correlation, and total energies (in hartrees) from various schemes of computations. The exact values are computed with HF densities.

Molecules	*Kinetic*		*Exchange*		*Correlation*		*Exch.-corr.*		*Total energy*	
	*T*_0_ ♣	*T*_0_+*T*_2_♣	*K* *^exact^* ♣	*K* *^PBE^* ♣	*E* *_c_* *^VWN^* ♦	*E* *_c_* *^GCP^* ♦	*E* *_xc_* *^exact^* ♣	*E* *_xc_* *^PBE^* ♣	*E* *_tot_* *^BLYP^* ♥	*E* *_tot_* *^TH^* ♥
**H** **_2_**	1.140	1.125	−0.657	−0.648	−95·10^−3^	−47·10^−3^	−0.698	−0.691	−1.169	−1.178
**LiH**	7.978	8.003	−2.125	−2.105	−219·10^−3^	−93·10^−3^	−2.212	−2.188	−8.068	−8.070
**CH** **_4_**	40.050	40.141	−6.576	−6.536	−593·10^−3^	−328·10^−3^	−6.883	−6.836	−40.502	−40.515
**H** **_2_** **O**	76.150	75.477	−8.910	−8.917	−664·10^−3^	−365·10^−3^	−9.292	−9.241	−76.448	−76.433
**HF**	100.137	99.242	−10.378	−10.385	−704·10^−3^	−380·10^−3^	−10.779	−10.720	−100.48	−100.455
**N** **_2_**	109.115	108.242	−13.094	−13.128	−945·10^−3^	−506·10^−3^	−13.665	−13.580	−109.559	−109.54
**O** **_2_**	149.843	148.369	−16.290	−16.358	−1110·10^−3^	−599·10^−3^	−16.958	−16.887	−150.384	−150.337
**F** **_2_**	198.892	196.729	−19.872	−19.951	−1302·10^−3^	−697·10^−3^	−20.661	−20.564	−199.599	−199.533

♣ : from Ref. [97];

♦ : from Ref. [101];

♥ : from Ref. [110]

**Table V.** Values (in hartrees) of the structural indices electronegativity (χ), chemical hardness (η), compute by means of the group method [14, 125] within the finite-difference [121, 122], density functional [123], and semiclassical [124] modes of computations for molecules of Table IV.

Level Molecule s	*Finite-Difference*		*Functional*		*Semiclassical*	
	*χ* *_FD_*	*η* *_FD_*	*χ* *_DFT_*	*η* *_DFT_*	*χ* *_SC_*	*η* *_SC_*
**H** **_2_**	0.26387	0.2370	0.26384	0.23704	0.26387	0.23705
**LiH**	0.15626	0.192	0.19212	0.12818	0.00811	0.006596
**CH** **_4_**	0.25616	0.2239	0.32216	0.29051	0.08468	0.03064
**H** **_2_** **O**	0.26871	0.2331	0.39097	0.34859	0.09335	0.0229
**HF**	0.3077	0.2479	0.51964	0.44974	0.08493	0.01639
**N** **_2_**	0.25616	0.2789	5.79566	4.91363	0.03139	0.00761
**O** **_2_**	0.27894	0.2257	10.6505	5.91694	0.04072	0.00816
**F** **_2_**	0.36898	0.2598	16.9129	4.37707	0.05061	0.00849

**Table VI.** Coefficients in bilinear correlation of the energies of Tables I and II against the electronegativity and chemical hardness of Table III within experimental finite difference (FD), density functional theory (DFT), and semiclassical (SC) modes of computations, respectively. The deviation from the parabolic expansion *E*=*a*+*b*χ+*c*η in terms of the index σ_π_ =sign(*b*) *c/b*^2^ as well as the correlation factor of the QSPR model (*r*) are both calculated. The color code indicates: the (+, +) for magenta background and (−, +) for green background sign combinations of (*b*, *c*) in (191), respectively, while the grey background is used in highlighting the representative (σ_*π*_, *r*) couple for each energy type of employed functionals.

Method of		QSPR results					Method of		QSPR results				
Energ y	(χ, η)	a	b	c	σ_π_	r	Energy	(χ, η)	a	b	c	σ_π_	r
***T*_*exact*_**	***FD***	194.59	741.55	−951.38	−0.0017	0.33	***E*_*xc*_^*exact*^**	***FD***	−15.30	−44.80	60.89	−0.0303	0.37
	***DFT***	51.73	2.09	31.51	7.2	0.62		***DFT***	−6.56	−0.19	−1.53	40.84	0.58
	***SC***	186.38	−2315.7	5147.4	−0.001	0.35		***SC***	−13.76	73.07	−128.0	−0.024	0.38
***T*_0_**	***FD***	179.67	687.26	−881.14	−0.0019	0.33	***E*_*xc*_^*I(Xα)*^**	***FD***	−15.28	−44.56	60.69	−0.0306	0.37
	***DFT***	46.99	1.91	29.41	8.07	0.62		***DFT***	−6.56	−0.2	−1.51	39.11	0.57
	***SC***	172.42	−2185.3	4874.1	−0.001	0.35		***SC***	−13.72	72.04	−125.5	−0.024	0.38
***T*_0_+*T*_2_**	***FD***	193.83	736.94	−946.29	−0.0017	0.33	***E*_*xc*_^*II(Xα)*^**	***FD***	−15.28	−44.81	60.92	−0.0303	0.37
	***DFT***	51.59	2.07	31.36	7.33	0.62		***DFT***	−6.52	−0.19	−1.53	40.81	0.57
	***SC***	185.59	−2312.1	5142	−0.001	0.35		***SC***	−13.75	74.72	−132.4	−0.024	0.38
***T*_*Padé*_**	***FD***	194.61	741.17	−951.43	−0.0017	0.33	***E*_*xc*_^*I(Wig)*^**	***FD***	−15.29	−44.53	60.67	−0.0306	0.37
	***DFT***	51.54	2.08	31.58	7.3	0.62		***DFT***	−6.58	−0.2	−1.50	38.78	0.57
	***SC***	186.42	−2334.4	5197.4	−0.001	0.35		***SC***	−13.72	71.4	−123.8	−0.024	0.38
***K*_*exact*_**	***FD***	−14.91	−43.62	59.38	−0.0312	0.37	***E*_*xc*_^*II(Wig)*^**	***FD***	−15.30	−44.80	60.95	−0.0304	0.37
	***DFT***	−6.37	−0.19	−1.49	43.17	0.57		***DFT***	−6.54	−0.2	−1.52	39.98	0.57
	***SC***	−13.4	72.74	−128.78	−0.0243	0.38		***SC***	−13.76	74.1	−130.7	−0.024	0.38
***K*_*0*_**	***FD***	−13.59	−40.29	54.69	−0.0337	0.37	***E*_*tot*_^*exact*^**	***FD***	−194.98	−742.7	952.91	−0.0017	0.33
	***DFT***	−5.72	−0.17	−1.39	46.96	0.58		***DFT***	−51.91	−2.10	−31.54	7.15	0.62
	***SC***	−12.24	68.77	−123.42	−0.026	0.38		***SC***	−186.73	2316	−5148	−0.001	0.35
***K*^*B88*^**	***FD***	−14.89	−43.61	59.33	−0.0312	0.37	***E*_*tot*_^*xc(RG)*^**	***FD***	−195.55	−743.9	954.5	−0.0017	0.33
	***DFT***	−6.37	−0.19	−1.49	42.35	0.57		***DFT***	−52.27	−2.11	−31.56	7.1	0.62
	***SC***	−13.39	71.88	−126.54	−0.0245	0.38		***SC***	−187.24	2315.	−5143.	−0.001	0.35
***E*_*c*_^*exact*^**	***FD***	−0.39	−1.20	1.52	−1.055	0.38	***E*_*tot*_^*xc(LDA)*^**	***FD***	−194.26	−740.7	950.13	−0.0017	0.33
	***DFT***	−0.19	−0.0075	−0.03	595.98	0.59		***DFT***	−51.59	−2.09	−31.47	7.18	0.62
	***SC***	−0.362	0.232	0.997	18.528	0.38		***SC***	−186.1	2313.	−5142.	−0.001	0.35
***E*_*c*_^*(139)*^♠**	***FD***	−0.40	−1.13	1.55	−1.207	0.39	***E*_*tot*_^*BLYP*^**	***FD***	−194.99	−742.7	952.86	−0.0017	0.33
	***DFT***	−0.19	−0.0056	−0.03	1081.3	0.57		***DFT***	−51.94	−2.1	−31.53	7.15	0.62
	***SC***	−0.356	0.851	−0.563	−0.778	0.41		***SC***	−186.74	2315.	−5145	−0.001	0.35
***E*_*c*_** •	***FD***	−0.41	−1.10	1.44	−1.1808	0.40	***E*_*tot*_^*PW91*^**	***FD***	−194.97	−742.7	952.81	−0.0017	0.33
	***DFT***	−0.22	−0.0063	−0.03	735.5	0.59		***DFT***	−51.92	−2.1	−31.54	7.15	0.62
	***SC***	−0.374	−0.224	2.113	−42.24	0.42		***SC***	−186.7	2315.	−5145.	−0.001	0.35

♠ ,

• : from associate energetic atomic values of Table I

**Table VII.** Coefficients in bilinear correlation of the energies of Table IV against the respective electronegativity and chemical hardness of the Table V. The computational meaning of the output values and the color code are the same as those of Table VI caption.

Method of		QSPR results					Method of		QSPR results				
Energy y	χ & η	a	b	c	σ_π_	r	Energy y	χ & η	a	b	c	σ_π_	r
***T*_0_**	***FD***	−192.71	821.61	238.59	0.00035	0.77	***T*_0_+*T*_2_**	***FD***	−190.58	811.48	237.96	0.0004	0.77
	***DFT***	40.446	8.125	4.501	0.068	0.89		***DFT***	40.199	8.012	4.497	0.07	0.89
	***SC***	82.14	542.16	−977.9	−0.0033	0.56		***SC***	81.4	537.74	−969.47	−0.003	0.56
***K*^*exact*^**	***FD***	18.18	−70.1	−38.02	0.0077	0.74	***K*^*PBE*^**	***FD***	18.324	−70.48	−38.25	0.0077	0.74
	***DFT***	−5.234	−0.646	−0.805	1.93	0.89		***DFT***	−5.219	−0.65	−0.811	1.92	0.89
	***SC***	−9.78	−48.27	94.96	−0.04	0.61		***SC***	−9.81	−48.15	95.	−0.04	0.61
***E*_*c*_^*VWN*^**	***FD***	1.039	−4.06	−2.74	0.167	0.71	***E*_*c*_^*GCP*^**	***FD***	0.59	−2.27	−1.496	0.289	0.72
	***DFT***	−0.423	−0.035	−0.06	47.83	0.87		***DFT***	−0.225	−0.019	−0.033	94.08	0.86
	***SC***	−0.71	−3.13	6.22	−0.63	0.64		***SC***	**−0.36**	**−2.1**	**3.7**	**−0.88**	**0.64**
***E*_*xc*_^*exact*^**	***FD***	18.95	−72.63	−40.1	0.0076	0.74	***E*_*xc*_^*PBE*^**	***FD***	18.85	−72.45	−39.62	0.0075	0.74
	***DFT***	−5.457	−0.668	−0.846	1.897	0.89		***DFT***	−5.421	−0.666	−0.84	1.898	0.89
	***SC***	−10.19	−50.16	98.71	−0.039	0.61		***SC***	−10.13	−49.97	98.28	−0.04	0.61
***E*_*tot*_^*BLYP*^**	***FD***	193.12	−824.17	−238.96	0.00035	0.77	***E*_*tot*_^*TH*^**	***FD***	193.06	−823.8	−239.04	0.0004	0.77
	***DFT***	−40.67	−8.149	−4.514	0.068	0.89		***DFT***	−40.67	−8.145	−4.514	0.068	0.89
	***SC***	−82.48	−544.35	981.7	−0.003	0.56		***SC***	−82.46	−544.3	981.5	−0.003	0.56
